# Daytime napping and the incidence of Parkinson’s disease: a prospective cohort study with Mendelian randomization

**DOI:** 10.1186/s12916-024-03497-7

**Published:** 2024-08-13

**Authors:** Fabin Lin, Yisen Shi, Wenjing Song, Yanhong Weng, Xinyang Zou, Xuanjie Chen, Jiayi Zheng, Ke Chen, Qinyong Ye, Xilin Wu, Guoen Cai

**Affiliations:** 1 Department of Neurology, Center for Cognitive Neurology, Institute of Clinical Neurology, Fujian Medical University Union Hospital, 29 Xinquan Road, Fuzhou, 350001 China; 2 Fujian Institute of Geriatrics, Fujian Medical University Union Hospital, 29 Xinquan Road, Fuzhou, 350001 China; 3 Fujian Key Laboratory of Molecular Neurology, Fujian Medical University, 88 Jiaotong Road, Fuzhou, 350001 China; 4 Fujian Medical University, Fuzhou, 350001 China; 5 Department of Neurosurgery, Fujian Medical University Union Hospital, 29 Xinquan Road, Fuzhou, 350001 China

**Keywords:** Daytime napping, Parkinson’s disease, Mendelian randomization, UK Biobank

## Abstract

**Background:**

The causal relationship between daytime napping and the risk of Parkinson’s disease (PD) remains unclear, with prospective studies providing limited evidence. This study investigated the association between daytime napping frequency and duration and PD incidence and explored the causality relationship between this association by conducting Mendelian randomization (MR) analysis.

**Methods:**

This prospective cohort study included 393,302 participants, and accelerometer-measured daytime napping data were available only for 78,141 individuals. Cox proportional hazards regression was used to estimate the association between the daytime napping frequency and duration and the PD risk. The role of the systemic immune-inflammation index (SII) in the association between daytime napping frequency and PD risk was assessed through mediation analyses. Moreover, the causal association between the daytime napping frequency and the PD risk was preliminarily explored by conducting two-sample MR analyses.

**Results:**

The median follow-up duration was 12.18 years. The participants who reported napping sometimes or usually exhibited a significantly higher PD risk than those who never/rarely napped during the day [sometimes: hazard ratio (HR), 1.13; 95% confidence interval (CI), 1.03–1.23; usually: HR, 1.33; 95% CI, 1.14–1.55], and SII played a mediating role in this association. However, the MR analyses did not indicate that the daytime napping frequency and PD risk were significantly associated. The participants napping for over 1 h exhibited a significantly elevated PD risk (HR, 1.54; 95% CI, 1.11–2.16). Moreover, no significant interaction was identified between napping frequency or duration and genetic susceptibility to PD (*P* for interaction > 0.05).

**Conclusions:**

In this study, increased daytime napping frequency and duration were associated with an increased PD risk, but no causal relationship was observed between napping frequency and PD risk in the MR analysis. Larger GWAS-based cohort studies and MR studies are warranted to explore potential causal relationships.

**Supplementary Information:**

The online version contains supplementary material available at 10.1186/s12916-024-03497-7.

## Background

Being the second most prevalent neurodegenerative disease worldwide, Parkinson’s disease (PD) is the fastest-growing neurological condition at the global scale in terms of morbidity, disability, and mortality since 1990 [1, 2]. Sleep disorder is among the most common non-motor symptoms of PD, occurring in 60–98% people diagnosed with PD. Sleep disorder is also frequently considered among the main causes of severe discomfort in patients [3]. Daytime napping has become a very common lifestyle habit, especially among elderly people [4]. Given the PD burden and popularity of daytime napping, determining the association between daytime napping and PD and developing preventive interventions are crucial public health concerns.

Few prospective studies have examined the association between daytime napping and PD risk, and their findings have been inconsistent. The Honolulu-Asia Aging Study, which included 3078 older adult men, reported no association between daytime napping and PD risk [5]. However, some other studies have drawn the opposite conclusion. For example, in a study based on osteoporotic fractures in men, objective prolonged napping was associated with an increased PD risk in older men [6]. Moreover, a cross-sectional study suggested that daytime napping is significantly associated with PD in older adult women [7]. In another study analyzing an older US population, longer daytime naps were associated with higher odds of PD [8]. However, considering the presence of study design limitations or population generalizability issues, further studies are warranted to assess this association.

By using self-reported and accelerometer-measured napping data from a large-scale population-based UK Biobank study, this study explored the relationship between daytime napping frequency and PD incidence through prospective observational analysis and Mendelian randomization (MR). MR analysis leverages genetic variants that affect modifiable risk factors to infer causal relationships between these exposures and health outcomes. This method is generally regarded as more robust against confounding factors and reverse causation compared to traditional observational approaches [9]. We also investigated, based on the polygenic risk score (PRS), whether daytime napping was associated with disease onset in a PD-susceptible population.

## Methods

### Study population

Between 2006 and 2010, the UK Biobank recruited approximately 500,000 UK residents who were aged 37–73 years at the time of recruitment. These participants provided regular blood, urine, and saliva samples and details about their lifestyles for analysis. These data were then linked to their health-related records. The UK Biobank conducted three major resurveys. The first resurvey was conducted in 2012–2013 and involved approximately 20,000 participants, the second resurvey started in 2014 and involved approximately 65,000 participants, and the third resurvey began in 2019 and involved approximately 5000 participants. This prospective cohort study was approved by the UK North West Multi-Centre Research Ethics Committee. Written informed consent was obtained from all participants [10]. The study was conducted in accordance with the Strengthening the Reporting of Observational Studies in Epidemiology (STROBE) reporting guidelines. Fig. S1 in the Additional File 2 presents the detailed participant inclusion and exclusion process in this study.

### Assessment of exposure

The daytime napping frequency was determined based on the participant’s response to the question “Do you have a nap during the day?” presented on a touch screen questionnaire. The response options included were never/rarely, sometimes, usually, and prefer not to answer. The option “Prefer not to answer” was categorized as missing data. The stability of the napping exposure frequency was verified using the Cochran-Mantel–Haenszel test [11]. The daytime napping duration was determined using the wrist-worn accelerometer data (Field 40030), which provided average sleep rates during each period. The sleep state was calculated using a combination of data sources and methodologies [12]. To deduce sleep state, the participants were made to wear a wrist-worn triaxial accelerometer (Axivity AX3) for capturing tri-axial acceleration data at 100 Hz [13–15]. On the study measurement day, a Vicon autographer wearable camera was worn by the participants during wakeful periods. The camera took photos every approximately 20 s [16]. Annotated images from the wearable camera were employed to construct a groundtruth of reference behaviors, including sleep. Sleep information was obtained from a simple sleep diary and a HETUS time-use diary. The groundtruth resulted in 213 activity labels, which were condensed into six free-living behavior labels, including “sleep.” Accelerometer data were preprocessed using standard procedures, including device calibration, resampling to 100 Hz, and removal of noise and gravity [17]. Features were extracted from 30-s time windows, which resulted in a 126-dimensional feature vector. Random forests (RFs) were used to classify the activities, thereby providing predictions based on an aggregate of individual CART trees. Balanced RFs were employed to account for the unbalanced dataset [18]. RFs classify data points but have a dearth of understanding of the temporal structure. A hidden Markov model (HMM) was used for time smoothing by encoding the temporal sequence of classes [19]. The Viterbi algorithm was applied to identify the most likely sequence of true activity states given observed emissions, thus correcting erroneous predictions. The HMM, which incorporated the temporal structure, was used to infer the most likely sequence of true activity states. The state of sleep, a specific behavior label, was deduced using the corrected sequence of predicted classes. The model trained on free-living groundtruth data was used to predict behavior in a large health dataset (UK Biobank) with 103,712 participants. The probability of engaging in specific behavior types, including sleep, was expressed for each 30-s epoch. In summary, the sleep state was deduced by collecting information from wearable cameras, sleep diaries, and accelerometer data. Using machine learning, including RFs and HMMs, helped to improve the accuracy of sleep state prediction and correct errors in initial classifications. We used 9 a.m. and 6 p.m. as the start and end times for daytime napping because our study included elderly participants and who are known to have phase advance (earlier bedtime) [20]. Additionally, we avoided potential morning and evening transition periods (7 a.m. to 9 a.m. and 6 p.m. to 8 p.m.). To eliminate interference from participants’ main sleep periods during the day, the habitual sleep/wake times were calculated for the individuals. Specifically, sleep scores were presented for each period based on the weighted sum of the current period, four preceding periods, and two subsequent periods. The calculation involves determining whether the sum is smaller than 1 h (indicating sleep) or not (indicating wakefulness). This methodology has been described in studies related to the UK Biobank database [21].

### Systemic immune-inflammation index

Peripheral blood samples collected from the UK Biobank participants were analyzed by the UK Biobank Central Laboratory within 24 h of sampling. The Beckman Coulter LH750 Hematology Analyzer was used to examine the blood cell samples in 4 mL of ethylenediaminetetraacetic acid vacuole (details at https://biobank.ndph.ox.ac.uk/ukb/ukb/docs/haematology.pdf.). The systemic immune-inflammation index (SII) was calculated using the following formula: SII = (neutrophil count * platelet count)/lymphocyte count [22].

### Assessment of outcomes

As recommended by the UK Biobank and previous studies [23, 24], algorithmically defined outcomes were used for determining PD onset in the cohort participants. Disease-related information was gathered by reviewing inpatient electronic health records and death registers linked to the Hospital Episode Statistics England, Scottish Morbidity Records, and Patient Episode Database for Wales on October 31, 2022 (England), July 31, 2021 (Scotland), and February 28, 2018 (Wales), respectively. Follow-up lasted from baseline to the time of PD diagnosis, death, loss to follow-up, or review, whichever occurred first.

### Polygenic risk score

The polygenic risk score (PRS) represents the correlation between the genotype and the PD risk. We here used the standard PRS for PD released by the UK Biobank (Field ID: 26,260), which was extracted from an external GWAS meta-analysis dataset, as described by Thompson et al. [25]; these authors had calculated PRS for 28 diseases and 25 quantitative traits. By using a Bayesian approach, the RPS algorithm was built based on trait-specific meta-analyses. An individual’s PRS was calculated as the genome-wide sum of posterior effect sizes per variant multiplied by the allelic dose. The raw PRS was centered and normalized to produce a corrected PRS for subsequent analyses.

### Two-sample MR

For determining the daytime napping frequency, 104 independent single nucleotide polymorphisms (SNPs) (*P* < 5 × 10^−8^, *r* ^2^ < 0.001, distance = 10,000 kb) from the GWAS study at the UK Biobank (*n* = 452,633) were used. These SNPs were replicated and validated in the 23andMe cohort (*n* = 541,333) [26]. The daytime napping category (never, sometimes, or usually) was considered a continuous variable in the MR analysis. Genotyping, quality control, and interpolation procedures for the UK Biobank data have been described elsewhere [27].

The GWAS summary data for PD were obtained from GWAS Catalog (https://www.ebi.ac.uk/gwas/home) and included 42,792 PD patients and 568,693 controls (Study ID: GCST90275127) [28].

Valid instrumental variables (IVs) had to satisfy the following three assumptions: (1) association with the risk exposure of interest (relevance), (2) no common cause with the outcome (independence), (3) affect the outcome only through the risk exposure (exclusion restriction). To ensure the reliability of the findings, the PLINK clumping method with a stringent clumping threshold (*r* ^2^ < 0.001, LD distance = 10,000 kb) was applied. This ensured that SNPs in the residual linkage-disequilibrium (LD) within a particular window were pruned to evaluate the bias caused by the residual LD of genetic variants. For harmonization, the strand for non-palindromic SNPs was corrected. For palindromic SNPs, rather than excluding them from the analysis, an attempt was made to infer the alleles on the positive strand using effect allele frequencies. Additionally, we estimated the F-statistic for instrumental variables, and the mean of *F* > 10 is generally sufficient for the MR analysis [29, 30]. The Mendelian randomization study followed the STROBE-MR guidelines [31, 32]. The STROBE-MR checklist can be found in Additional File 1.

### Statistical analyses

Hazard ratios (HRs) and 95% confidence intervals (CIs) for the UK Biobank-based observational analysis were estimated using the Cox proportional hazards model. The group of participants who never napped was used as a reference for this estimation. We reported the results based on three models: (1) unadjusted; (2) adjusted for age and sex; (3) adjusted for age, sex, ethnicity, body mass index (BMI), household income, education, smoking status, alcohol intake frequency, total hours of physical activity per week, family history of PD, and other sleep-related covariates (Additional File 2: Text. S1). The association between daytime napping and the PD risk in various genetic risk groups was determined using a cut-off point stratified by PRS tertiles for PD. The interaction between PRS and daytime napping in their effect on PD development was investigated using likelihood ratio tests. A series of sensitivity analyses were conducted: (1) excluding PD events in the previous 2 or 4 years; (2) excluding self-reported PD outcomes; (3) excluding participants who reported working at night; (4) excluding participants who reported having sleep disorders; (5) including death as a competing risk through a competing risk model; (6) further correcting for difficulty in waking up at morning, chronotype, sleeplessness, snoring, and daytime dozing as sleep-related conditions. Using a regression-based approach, mediation analyses were performed to decompose the direct, indirect, and total effects between daytime napping and PD and to estimate the proportion of this SII-mediated association. In the mediation analyses, the effect of the exposure variable on the mediator variable was defined as “a,” the effect of the mediator variable on the outcome was defined as “b,” and the total effect of the exposure variable on the outcome was defined as “c.” According to previous studies [33], the effect of the exposure variable on the outcome through the mediating variable was “a*b.” Thus, the proportion of mediation was (a*b)/c. The number of boots applied for the mediation analyses was 200. The mediation analysis was conducted after adjustment for the confounding factors of age, gender, smoking status, alcohol consumption, BMI, and physical activity.

To completely examine the causal association between daytime naps and PD, a bidirectional two-sample MR was conducted. Two-sample MR methods, wherein exposure and outcome are measured in non-overlapping datasets, allow for minimal false-positive rates and increased sample sizes [34]. In the MR analysis, random-effects inverse variance weighting (IVW) was used as the primary method, and MR-Egger regression [35], weighted median models [36], simple mode, and weighted mode were used as secondary methods [37]. The Wald ratio test was used when < 3 SNPs were available for the analysis. To prevent any potential violations of MR assumptions, we tested for pleiotropy (through MR-Egger regression intercepts, and MR Pleiotropy Residual Sum and Outlier [MR-PRESSO] global test) and heterogeneity (based on Cochran’s *Q* values [38]).

In two-sample MR, we first performed preliminary analyses based on IVW and four other secondary analyses. In the sensitivity analysis, to avoid reverse causation, the Steiger test was conducted for each SNP so as to determine whether the exposure *R* ^2^ (the variance of the disease/trait explained by the selected SNP) was greater than the outcome *R* ^2^, excluding SNPs that tested “False” (outcome *R* ^2^ > exposure *R* ^2^) [39]. When pleiotropy or heterogeneity was identified (*P* < 0.05), then we applied MRPRESSO to identify and eliminate outliers until no more outliers were identified [40]. We then performed Radial-MR to determine whether outliers were present (the threshold was set at 0.05) [41], and, if outliers were present, they were eliminated and Radial-MR was repeated until no outliers were detected. After excluding the abovementioned SNPs, the analyses were reconducted.

Statistical analysis was conducted in R, Version 4.2.3. The MR analysis was conducted using the “TwoSampleMR” (Version 0.5.6) and “RadialMR” software package.

## Results

### Descriptive characteristics of the prospective cohort

This prospective cohort study involved 393,302 participants [mean age: 56.27 years; 186,812 males (47.5%)] and a median follow-up period of 12.18 (SD = 1.83) years. Of them, 224,646 (57.1%) participants reported they never/rarely took a daytime nap, 148,094 (37.7%) participants napped sometimes, and 20,562 (5.2%) participants usually napped during the day. Compared with those reporting to never/rarely take a daytime nap, the participants who napped sometimes or usually were more likely to be older, male, poorer, and current smokers; have a higher BMI; be non-drinkers; and have received lower levels of education (Table 1). Furthermore, the participants who found waking up in the morning difficult, who found being a “morning” person difficult, who had insomnia, who snored, and who had the habit of daytime dozing had a higher daytime napping frequency (Additional File 2: Table S1). The study investigated the daytime napping duration of 83,151 participants. Table S2 in Additional File 2 presents their baseline characteristics. The baseline characteristics of the 16,535, 54,769, and 4581 participants in the first, second, and third follow-up visits, respectively, are presented in Tables S3–S5 in Additional File 2.

**Table 1:** Baseline characteristics of participants for nap frequency

Characteristics		Daytime napping				*P-*value
		Overall	Never/rarely	Sometimes	Usually	
Participants, n		393302	224646	148094	20562	
Age, Mean(SD)		56.27 (8.11)	55.20 (8.11)	57.47 (7.92)	59.38 (7.53)	<0.001
Sex, n (%)	Female	206490 (52.5)	128615 (57.3)	71261 (48.1)	6614 (32.2)	<0.001
	Male	186812 (47.5)	96031 (42.7)	76833 (51.9)	13948 (67.8)	
Ethnicity, n (%)	White	360803 (91.7)	206781 (92.0)	135275 (91.3)	18747 (91.2)	<0.001
	Black	2413 (0.6)	948 (0.4)	1294 (0.9)	171 (0.8)	
	Asia	6632 (1.7)	3345 (1.5)	2794 (1.9)	493 (2.4)	
	Other	23454 (6.0)	13572 (6.0)	8731 (5.9)	1151 (5.6)	
BMI (kg/m2), Mean(SD))		27.30 (4.70)	26.83 (4.48)	27.85 (4.86)	28.52 (5.23)	<0.001
Household income, n (%)	Greater than 100,000 £	20800 (5.3)	14535 (6.5)	5804 (3.9)	461 (2.2)	<0.001
	18,000 £ to 100,000 £	256381 (65.2)	152000 (67.7)	92582 (62.5)	11799 (57.4)	
	Less than 18,000 £	73558 (18.7)	34639 (15.4)	32820 (22.2)	6099 (29.7)	
	Unknow	42563 (10.8)	23472 (10.4)	16888 (11.4)	2203 (10.7)	
Education, n (%)	Any school degree	46249 (11.8)	27809 (12.4)	16365 (11.1)	2075 (10.1)	<0.001
	College or University degree	138859 (35.3)	84795 (37.7)	47784 (32.3)	6280 (30.5)	
	Vocational	20452 (5.2)	11763 (5.2)	7799 (5.3)	890 (4.3)	
	Other	187742 (47.7)	100279 (44.6)	76146 (51.4)	11317 (55.0)	
Current tobacco smoking, n (%)	No	353469 (89.9)	204166 (90.9)	131604 (88.9)	17699 (86.1)	<0.001
	Only occasionally	10873 (2.8)	6068 (2.7)	4200 (2.8)	605 (2.9)	
	Yes, on most or all days	28960 (7.4)	14412 (6.4)	12290 (8.3)	2258 (11.0)	
Alcohol, n (%)	Never	15388 (3.9)	8101 (3.6)	6223 (4.2)	1064 (5.2)	<0.001
	Previous	13316 (3.4)	6350 (2.8)	5739 (3.9)	1227 (6.0)	
	Current	364598 (92.7)	210195 (93.6)	136132 (91.9)	18271 (88.9)	
Physical activity (minutes/week), Mean(SD)		2651.30 (2708.29)	2647.33 (2668.25)	2654.96 (2733.00)	2668.35 (2952.70)	0.457
Sleep duration (hours/day), Mean(SD)		7.16 (1.08)	7.07 (0.99)	7.23 (1.12)	7.69 (1.54)	<0.001
Family history of PD, n (%)	No	377120 (95.9)	215524 (95.9)	141917 (95.8)	19679 (95.7)	0.103
	Yes	16182 (4.1)	9122 (4.1)	6177 (4.2)	883 (4.3)	

*Abbreviation*: *BMI* body mass index, *PD* Parkinson’s disease, *SD* standard deviation

Data are presented as mean (standard deviation) or n (%). The one-way ANOVA tests were used for continuous variables and χ^2^ tests were used for categorical variables

### Association between daytime napping and PD risk

**Fig. 1:**
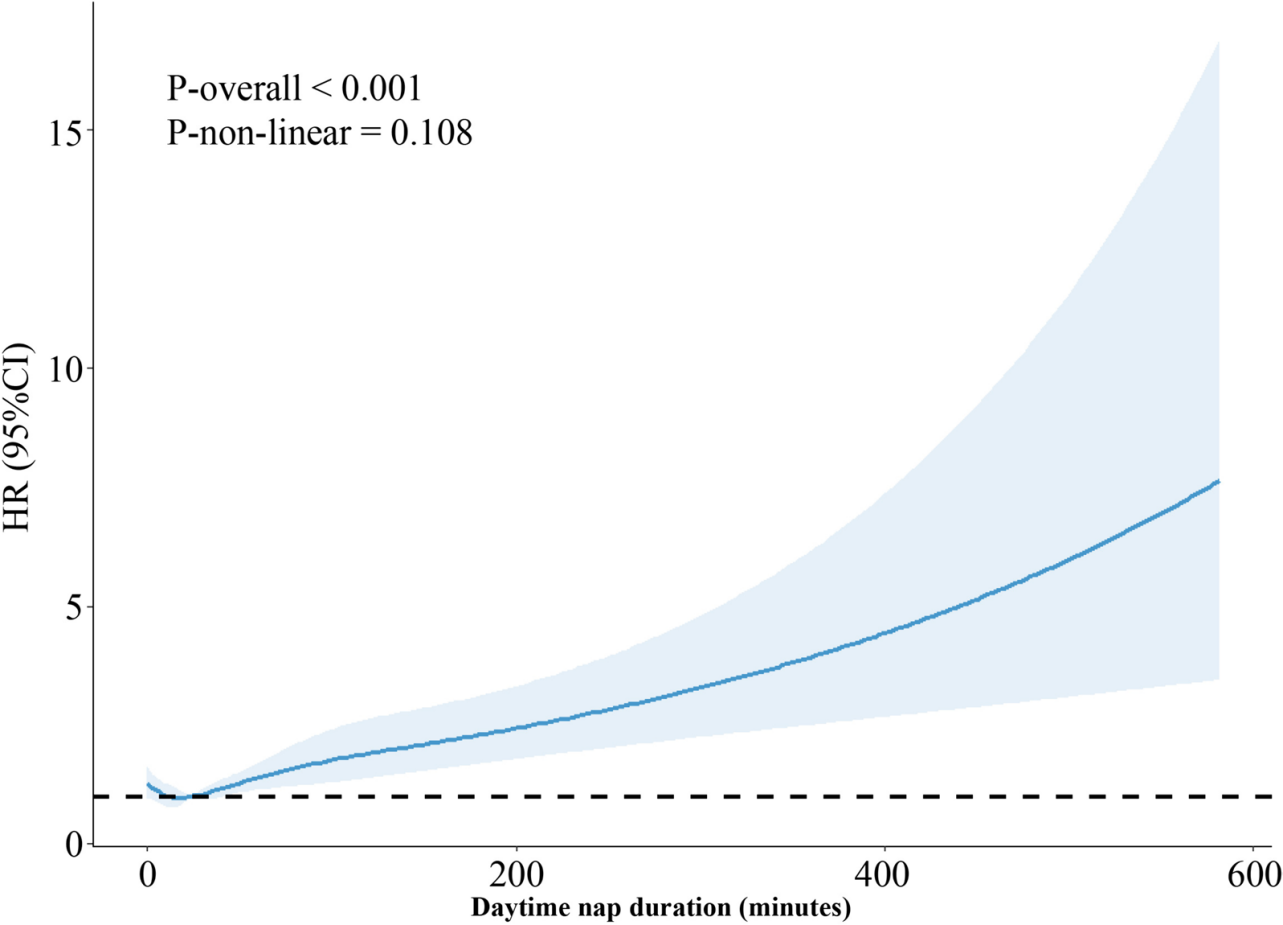
Multivariable adjusted dose–response associations between daytime napping duration and risk of incident Parkinson’s disease. Adjusted for age, sex, race, household income, education, BMI, current tobacco smoking, physical activity, alcohol, sleep duration, and family history of Parkinson’s disease

After a range of covariates were adjusted, a significantly increased PD risk was noted among the participants who reported taking a daytime nap sometimes or usually compared with among those who never/rarely took a daytime nap. The participants who reported napping sometimes had a 13% higher PD risk (HR, 1.13; 95% CI, 1.03–1.23; *P* = 0.009) compared with those reported never. The participants who reported frequent daytime napping had a 33% increased PD risk (HR, 1.33; 95% CI, 1.14–1.55; *P* = 0.001) compared with those reported never (Table 2). Similar results were observed when the three follow-up visits were assessed separately (Additional File 2: Table S6). The participants who napped less than an hour a day exhibited an increased PD risk, whereas those who napped over an hour a day displayed an 54% increased PD risk (HR, 1.54; 95% CI, 1.11–2.16; *P* = 0.01; Table 3). Afternoon naps were associated with an increased PD risk [13:00 pm–15:00 pm: 1.02 (1.01–1.03), *P* < 0.001, 16:00 pm–18:00 pm: 1.01 (1.00–1.02), *P* = 0.001], whereas morning naps are not associated with the PD risk (9:00 am–12:00 am: 1.00 (1.00–1.01), *P* = 0.107) (Additional File 2: Table S7). The dose–response analysis revealed a positive linear relationship between the daytime napping duration and the PD risk (Fig. 1). The results remained robust through the sensitivity analysis (Additional File 2: Table S8-S13).

**Table 2:** Association between daytime napping frequency and incidence of Parkinson’s disease (*N*=393302)

Characteristic	Daytime napping		
	Never/rarely	Sometimes	Usually
Participants, n (%)	224646 (57.1%)	148094 (37.7%)	20562 (5.2%)
person-years	2753818	1792200	243552
Cases	972	1010	223
Incident cases per 100 000 person-years	35.3	56.36	91.56
Models		HR (95%CI) *P*-value	HR (95%CI) *P*-value
Model 1	1.00 (Reference)	**1.61 (1.47-1.75) <0.001**	**2.63 (2.28-3.04) <0.001**
Model 2	1.00 (Reference)	**1.16 (1.06-1.26) 0.001**	**1.40 (1.21-1.63) <0.001**
Model 3	1.00 (Reference)	**1.13 (1.03-1.23) 0.009**	**1.33 (1.14-1.55) <0.001**

*Abbreviation*: *HR* hazard ratio, *BMI* body mass index

Model 1: unadjusted

Model 2: age, sex

Model 3: age, sex, race, household income, education, BMI, current tobacco smoking, physical activity, alcohol, Sleep duration, and family history of Parkinson’s disease

**Table 3:** Association between daytime napping during and incidence of Parkinson’s disease (*N*=78141)

Characteristic	Daytime napping			
	0 hour	<1 hour	≥1 hour	Continues
Participants, n (%)	20837 (26.7%)	43914 (56.2%)	13390 (17.1%)	
person-years	258844	560511	165227	
Cases	70	131	75	
Incident cases per 100 000 person-years	27	23	45	
Models		HR (95%CI) *P*-value	HR (95%CI) *P*-value	HR (95%CI) *P*-value
Model 1	1.00 (Reference)	0.89 (0.67-1.19) 0.430	**1.69 (1.22-2.34) <0.001**	**1.01 (1.00-1.01) <0.001**
Model 2	1.00 (Reference)	0.87(0.65-1.17) 0.350	**1.48 (1.07-2.05) 0.020**	**1.01 (1.00-1.01) <0.001**
Model 3	1.00 (Reference)	0.89 (0.66-1.19) 0.431	**1.54 (1.11-2.16) 0.01**	**1.01 (1.00-1.01) <0.001**

*Abbreviation*: *HR* hazard ratio, *BMI* body mass index

Model 1: unadjusted

Model 2: age, sex

Model 3: age, sex, race, household income, education, BMI, current tobacco smoking, physical activity, alcohol, sleep duration, and family history of Parkinson’s disease

### Joint association between the PRS, daytime napping, and PD

No significant interaction was observed between the daytime napping duration and PRS in their effect on PD development (*P* for interaction = 0.178; Table 4). Similarly, no interaction was observed between the daytime napping frequency and PRS in their effect on PD occurrence (*P* for interaction = 0.225).

**Table 4:** Risk of incident Parkinson’s disease according to daytime napping status stratified by PRS^a^ (*N*=384242 and 78141)

Daytime napping		PRS			*P* value for interaction^b^
		Low	Intermediate	High	
		HR (95%CI) *P*-value	HR (95%CI) *P*-value	HR (95%CI) *P*-value	
Frequency (*N*=384242)	Never/rarely	1.00 (Reference)	1.00 (Reference)	1.00 (Reference)	0.225
	Sometimes	1.2 (0.99-1.46, *p*=0.065)	1.05 (0.89-1.23, *p*=0.594)	**1.15** **(1.01-1.31, ** ***p*** **=0.041)**	
	Usually	1.28 (0.92-1.77, *p*=0.143)	**1.51** **(1.17-1.96, ** ***p*** **=0.002)**	1.2 (0.95-1.52, *p*=0.125)	
Duration (*N*= 78141)	0 hour	1.00 (Reference)	1.00 (Reference)	1.00 (Reference)	0.178
	1 hour	1.17 (0.61-2.23) 0.63	0.73(0.42-1.26) 0.25	0.89 (0.59-1.34) 0.59	
	≥1 hour	1.96 (0.96-4.03) 0.07	1.00 (0.51-1.96) 0.99	**1.76** **(1.11-2.80) 0.02**	

*Abbreviation*: *HR* hazard ratio, *BMI* body mass index

^a^Model adjusted for age, sex, race, household income, education, BMI, current tobacco smoking, physical activity, alcohol, sleep duration, and family history of Parkinson’s disease

^b^ *P* value for the interaction between daytime napping and polygenic risk score

### MR analysis

Table S14 in the Additional File 2 presents the two-sample MR results before excluding the outliers identified by MRPRESSO or Radial-MR. The F-statistics for individual SNPs were presented in Table S15 in Additional File 2. Regarding the impact of daytime napping on the incidence of PD, the random-effects IVW model showed no significant association between them (OR, 0.816, 95% CI, 0.510 to 1.304). This result remained consistent in other models. No heterogeneity or horizontal pleiotropy was detected (*P* > 0.05). With PD as exposure, the random-effects IVW model displayed no significant association between genetic liability to PD and daytime napping (*β*, 0.008, 95% CI, − 0.006 to 0.022). However, IVW and MR-Egger revealed the presence of heterogeneity and the MRPRESSO global test displayed the presence of horizontal pleiotropy (*P* < 0.05). In the sensitivity analysis, SNPs that did not pass the Steiger test, and outliers identified by MRPRESSO or Radial-MR have been displayed in Table S16 in Additional File 2. After the outliers were removed, no significant heterogeneity or horizontal pleiotropy was observed (Additional File 2: Table S17). The random-effects IVW indicated that no significant association between genetic liability to PD and daytime napping (*β*, − 0.003; 95% CI, − 0.008 to 0.002). This result was consistent across other models. Moreover, the leave-one-out method was employed to test the stability of the results (Additional File 3: Fig. S2 and Additional File 4: Fig. S3).

### Mediating role of SII in the association between the daytime napping frequency and PD incidence

Fig. S4 in Additional File 2 presents the results of mediation analyses for the association between SII and PD occurrence. Each unit increase in SII (Ln-transformed) was associated with a 19.1% increase in the PD risk (HR, 1.191; 95% CI, 1.098–1.291). For the association between the daytime napping frequency and SII, the participants who reported napping sometimes (*β*, 0.015; 95% CI, 0.012–0.019) or usually (*β*, 0.033; 95% CI, 0.025–0.040) had significantly higher SII levels compared with those who reported never napping. The mediation analyses revealed that SII mediated 1.75% (95% CI, 0.83–4.12%) and 2.05% (95% CI, 1.02–4.81%) of the association in the effect of sometimes and usually napping, respectively, on PD development.

## Discussion

This large prospective cohort study found that the higher frequency and longer duration of daytime napping were associated with an increased PD risk. The MR analysis revealed no reciprocal causal relationship between the daytime napping frequency and the PD risk. Additionally, the higher frequency of daytime napping was associated with an increase in the immune inflammatory response, which elevated the PD risk. Moreover, no significant interaction was observed between the frequency and duration of daytime napping and PRS in their effect on the PD risk.

The observational study results align with those of numerous epidemiologic studies, indicating that the increased daytime napping frequency and longer nap duration are linked to a higher PD risk. In a multicenter prospective study, 2920 men without a history of PD were followed for 11 years. The study ultimately revealed 106 PD events and demonstrated a correlation between objectively prolonged napping and a higher PD risk in older men [6]. A multicenter cohort study of 2675 community-dwelling older women reported that both subjective and objective naps were associated with PD [7]. In a United States-based cohort study with 213,885 volunteers having no history of PD, subjective daytime napping was associated with a higher PD risk [8]. A previous study based on the Honolulu-Asia Aging Study revealed that subjective daytime napping is associated with the PD risk in men [5].

The MR analysis revealed no causal relationship between the daytime napping frequency and the PD risk. Nevertheless, caution is warranted in interpreting these findings because of the small sample size and low precision of our study. The analyses were also constrained by a rudimentary assessment of the daytime napping frequency through a questionnaire, which resulted in the lack of details on duration or timing [26]. Our attempts to partially validate the specificity of identified loci, transitioning from self-reporting of data to objective determination through accelerometers, may have faced limitations because of the phenotypic differences between self-report and accelerometer data. Additionally, the relatively small sample size in the accelerometer subsample and the time lag between measurements, with accelerometers worn between 2 and 10 years after the study baseline constrained the assessment. Moreover, additional larger studies using GWAS-based cohorts and MR approaches are necessary for thoroughly investigating the relationship between the daytime napping frequency and the PD risk.

A definitive physiological mechanism directly explaining the association between daytime napping and an elevated PD risk is lacking and remains a challenge. In a study, sleep was objectively measured in older adults from the community, not clinically diagnosed with PD, by using an activity recorder [42]. The study revealed that the degree of sleep fragmentation was associated with Lewy body pathological burden as well as nigrostriatal neuronal loss. The increased odds of developing PD were correlated with greater sleep fragmentation, which, in turn, was associated with Lewy body pathology burden and nigrostriatal neuron loss [43]. Sleep fragmentation can possibly contribute to PD pathology, with potential mechanisms involving oxidative stress promotion or impairment in toxic protein clearance. In model organisms, both sleep fragmentation and sleep deprivation are associated with brain oxidative stress, a factor linked to PD pathogenesis [44–46]. Moreover, napping might be an integral component of the PD pathological process. The suprachiasmatic nucleus (SCN), which plays a crucial role in regulating the sleep–wake cycle, may contribute to reduced melatonin secretion and sleep–wake disruption, particularly in elderly healthy individuals with decreased SCN activity [47, 48]. Animal model studies have revealed abnormal electrical activity in the SCN of α-synuclein-overexpressing mouse models of PD [49]. Subsequent research should delve deeper into the underlying mechanisms.

The examination of the interaction between the daytime napping frequency or duration and the PRS on the PD risk yielded no significant results. This lack of significance suggests that genetic factors does not influence the association between daytime napping and the PD risk. These findings align with those of prior research. Benjamin et al. analyzed the interaction between PRS and a precursor symptom of PD, “sleepiness” [50]. The results of this analysis unveiled no significant interaction, further confirming that genetic factors are not substantially involved in the relationship between daytime napping and the PD risk.

Higher daytime napping frequency and longer daytime napping duration increased the PD risk by increasing the immune inflammatory response. Considering that daytime napping may be associated with an increase in the levels of inflammatory markers, the effect of napping on PD was hypothesized to be related to the inflammatory response [51]. A series of cross-sectional studies were conducted in a community-based population in China. Longer daytime naps were correlated to higher IL-6 levels. Inflammatory cytokines may be served as a crucial link between daytime napping and nonalcoholic fatty liver disease. While inflammation and immune dysfunction impair neuronal health and survival and are associated with PD, including both the motor and non-motor components of PD [52]. A meta-analysis included 152 studies and extracted studies reporting concentrations of blood or cerebrospinal fluid (CSF) markers in PD patients and controls. Significant alterations in the levels of inflammatory markers were observed between the PD group and controls. Increased IL-6, TNF-α, IL-1β, STNFR1, CRP, CCL2, CX3CL1, and CXCL12 levels and decreased INF-γ and IL-4 levels were observed in the PD group. In addition, increased CSF levels of IL-6, TNF-α, IL-1β, CRP, and CCL2 were observed in the PD group compared with the control group [53].

The direction of the relationship is a key issue in the study of sleep and PD. Thus, benefiting from the long follow-up of the UK Biobank study, we found similar results when PD cases were excluded at risk 2 years and 4 years after the baseline, a method reported in previous studies [6].

## Limitations

This study has some limitations. First, although we adjusted for most known PD-associated confounding variables, some unknown confounders may have exerted their effects. Second, the latency period for the rapid eye movement sleep behavior disorder as a pre-Parkinsonian symptom can be up to 10 years. Although our sensitivity analysis excluded patients with sleep disorders, bias may have been introduced by the occult population. Third, a fundamental assumption of MR is that SNPs are not linked to any confounders of the exposure or the outcome. Even when pleiotropic bias is taken into account, no MR study can entirely eliminate the possibility of pleiotropic bias. Finally, the UK Biobank participants were predominantly Europeans. Further studies are warranted to investigate the extent to which these findings can be applied to other populations.

## Conclusions

The results of this large UK Biobank-based cohort study suggested that the increased frequency of self-reported daytime naps and duration of accelerometer measurements are associated with an increased PD risk. The MR analysis provided no evidence that the frequency of daytime naps was associated with PD outcomes. Further larger GWAS-based cohort and MR studies are warranted to validate the findings.

### Supplementary Information

Additional file 1. STROBE-MR checklist.

Additional file 2: Text S1, Table S1-S15, and Figures S1, S3. Text S1- [Assessment of covariates]. Table S1- [Baseline sleep characteristics of participants based on frequency of daytime naps]. Table S2- [Baseline characteristics of participants based on daytime napping during]. Table S3- [Baseline characteristics of participants based on daytime napping frequency (First repeat assessment visit (2012–2013))]. Table S4- [Baseline characteristics of participants based on daytime napping frequency (Imaging visit (2014 +))]. Table S5- [Baseline characteristics of participants based on daytime napping frequency (First repeat imaging visit (2019 +))]. Table S6- [Daytime nap frequency and Parkinson’s disease prevalence when using different time points as a baseline]. Table S7- [Relationship between daytime napping duration and onset of Parkinson’s disease when napping was taking place at different times (N = 78,141)]. Table S8- [Association between daytime napping and incident Parkinson’s disease after excluding participants who experienced an outcome event within the first two years of follow-up]. Table S9- [Association between daytime napping and incident Parkinson’s disease after excluding participants who experienced an outcome event within the first four years of follow-up]. Table S10- [Association between daytime napping and incident Parkinson’s disease after excluding participants who self-reported Parkinson’s disease]. Table S11- [Association between daytime napping and incident Parkinson’s disease after excluding participants who worked night shifts]. Table S12- [Association between daytime napping and incident Parkinson’s disease after excluding participants with sleep disorders]. Table S13- [Associations between daytime napping and incident Parkinson’s disease by treating all-cause death as a competing risk]. Table S14- [Analysis of the association between daytime napping and PD with bidirectional two-sample Mendelian randomization]. Table S15- [Outliers identified in MR-PRESSO and Radial-MR]. Fig S1- [Flow chart of the screening process for this study]. Fig S4- [Mediation analysis of the role of systemic immune-inflammation index (SII) in the association between nap frequency and the development of PD].

Additional file 3: Fig S2- [(A) Scatter plots of genetic associations with daytime napping against the incidence of PD. (B) Funnel plot to assess heterogeneity. The blue line represents the inverse-variance weighted estimate, and the dark blue line represents the Mendelian randomization-Egger estimate. (C) Forest plot of the causal effects of single nucleotide polymorphisms associated with daytime napping on PD. The red lines are MR results of MR-Egger test and IVW method. (D) MR leave-one-out sensitivity analysis for daytime napping frequency on PD. Each black point represents the IVW-MR method applied to estimate the causal effect of daytime napping frequency on PD excluding that particular variant from the analysis].

Additional file 4: Fig S3- [(A) Scatter plots of genetic associations with genetic liability to PD on daytime napping. (B) Funnel plot to assess heterogeneity. The blue line represents the inverse-variance weighted estimate, and the dark blue line represents the Mendelian randomization-Egger estimate. (C) Forest plot of the causal effects of single nucleotide polymorphisms associated with genetic liability to PD on daytime napping. The red lines are MR results of MR-Egger test and IVW method. (D) MR leave-one-out sensitivity analysis for genetic liability to PD on daytime napping. Each black point represents the IVW-MR method applied to estimate the causal effect of genetic liability to PD on daytime napping excluding that particular variant from the analysis].

## Data Availability

All data used for Mendelian randomization analyses are publicly available from the respective GWAS. Cohort data from UK Biobank are available after the application (https://www.ukbiobank.ac.uk/).

## Abbreviations

BMI: Body mass index
CIs: Confidence intervals
CSF: Cerebrospinal fluid
GWAS: Genome-wide association study
HMM: Hidden Markov model
HRs: Hazard ratios
IVW: Inverse variance weighting
LD: Linkage disequilibrium
MR: Mendelian randomization
MRPRESSO: MR pleiotropy residual sum and outlier
PD: Parkinson’s disease
PRS: Polygenic risk score
RFs: Random forests
SCN: Suprachiasmatic nucleus
SII: Systemic immune-inflammation index
SNPs: Single nucleotide polymorphisms
STROBE: Strengthening the Reporting of Observational Studies in Epidemiology
