# Supplementary material for: Daytime napping and the incidence of Parkinson’s disease: a prospective cohort study with Mendelian randomization
Source: BMC Med. 2024 Aug 13;22:326. doi: 10.1186/s12916-024-03497-7 (PMC11321229; doi:10.1186/s12916-024-03497-7)
Supplement: Supplementary file 1 — Additional file 1. STROBE-MR checklist. [file 12916_2024_3497_MOESM1_ESM.docx]

**STROBE-MR checklist of recommended items to address in reports of Mendelian randomization studies**^1^ ^2^

| **Item No.** | **Section** | **Checklist item** | **Page No.** | **Relevant text from manuscript** |
| --- | --- | --- | --- | --- |
| 1 | **TITLE and ABSTRACT** | Indicate Mendelian randomization (MR) as the study’s design in the title and/or the abstract if that is a main purpose of the study | 1 | Daytime napping and the incidence Parkinson’s disease: A prospective cohort study with Mendelian randomization |
|  | **INTRODUCTION** |  |  |  |
| 2 | **Background** | Explain the scientific background and rationale for the reported study. What is the exposure? Is a potential causal relationship between exposure and outcome plausible? Justify why MR is a helpful method to address the study question | 2, 3 | Sleep disorder is among the most common non-motor symptoms of PD, occurring in 60%–98% people diagnosed with PD. Sleep disorder is also frequently considered among the main causes of severe discomfort in patients. Daytime napping has become a very common lifestyle habit, especially among elderly people. Given the PD burden and popularity of daytime napping, determining the association between daytime napping and PD and developing preventive interventions are crucial public health concerns.  The Honolulu-Asia Aging Study, which included 3,078 older adult men, reported no association between daytime napping and PD risk. However, some other studies have drawn the opposite conclusion. For example, in a study based on osteoporotic fractures in men, objective prolonged napping was associated with an increased PD risk in older men. Moreover, a cross-sectional study suggested that daytime napping is significantly associated with PD in older adult women. In another study analyzing an older US population, longer daytime naps were associated with higher odds of PD. However, considering the presence of study design limitations or population generalizability issues, further studies are warranted to assess this association. |
| 3 | **Objectives** | State specific objectives clearly, including pre-specified causal hypotheses (if any). State that MR is a method that, under specific assumptions, intends to estimate causal effects | 3 | MR analysis leverages genetic variants that affect modifiable risk factors to infer causal relationships between these exposures and health outcomes. This method is generally regarded as more robust against confounding factors and reverse causation compared to traditional observational approaches |
|  | **METHODS** |  |  |  |
| 4 | **Study design and data sources** | Present key elements of the study design early in the article. Consider including a table listing sources of data for all phases of the study. For each data source contributing to the analysis, describe the following: | 5 | For determining the daytime napping frequency, 94 independent single nucleotide polymorphisms (SNPs) (P < 5×10−8, r2 < 0.001, distance = 10,000 kb) from the GWAS study at the UK Biobank (n = 452,633) were used. These SNPs were replicated and validated in the 23andMe cohort (n = 541,333). The daytime napping category (never, sometimes, or usually) was considered a continuous variable in the MR analysis. Genotyping, quality control, and interpolation procedures for the UK Biobank data have been described elsewhere.  The GWAS summary data for PD were obtained from GWAS Catalog (https://www.ebi.ac.uk/gwas/home) and included 42,792 PD patients and 568,693 controls (Study ID: GCST90275127). |
|  | a) | Setting: Describe the study design and the underlying population, if possible. Describe the setting, locations, and relevant dates, including periods of recruitment, exposure, follow-up, and data collection, when available. |  | Does not apply |
|  | b) | Participants: Give the eligibility criteria, and the sources and methods of selection of participants. Report the sample size, and whether any power or sample size calculations were carried out prior to the main analysis | 5 | For determining the daytime napping frequency, 104 independent single nucleotide polymorphisms (SNPs) (P < 5×10−8, r2 < 0.001, distance = 10,000 kb) from the GWAS study at the UK Biobank (n = 452,633) were used. These SNPs were replicated and validated in the 23andMe cohort (n = 541,333)[26]. The daytime napping category (never, sometimes, or usually) was considered a continuous variable in the MR analysis. Genotyping, quality control, and interpolation procedures for the UK Biobank data have been described elsewhere[27].  The GWAS summary data for PD were obtained from GWAS Catalog (https://www.ebi.ac.uk/gwas/home) and included 42,792 PD patients and 568,693 controls (Study ID: GCST90275127)[28]. |
|  | c) | Describe measurement, quality control and selection of genetic variants | 5 | To ensure the reliability of the findings, the PLINK clumping method with a stringent clumping threshold (r2 < 0.001, LD distance = 10,000 kb) was applied. This ensured that SNPs in the residual linkage-disequilibrium (LD) within a particular window were pruned to evaluate the bias caused by the residual LD of genetic variants. |
|  | d) | For each exposure, outcome, and other relevant variables, describe methods of assessment and diagnostic criteria for diseases | 5 | The daytime napping category (never, sometimes, or usually) was considered a continuous variable in the MR analysis. Genotyping, quality control, and interpolation procedures for the UK Biobank data have been described elsewhere |
|  | e) | Provide details of ethics committee approval and participant informed consent, if relevant |  | Does not apply |
| 5 | **Assumptions** | Explicitly state the three core IV assumptions for the main analysis (relevance, independence and exclusion restriction) as well assumptions for any additional or sensitivity analysis | 5 | Valid instrumental variables (IVs) had to satisfy the following three assumptions: (1) association with the risk exposure of interest (relevance); (2) no common cause with the outcome (independence); (3) affect the outcome only through the risk exposure (exclusion restriction). |
| 6 | **Statistical methods: main analysis** | Describe statistical methods and statistics used | 6-7 | To completely examine the causal association between daytime naps and PD, a bidirectional two-sample MR was conducted. Two-sample MR methods, wherein exposure and outcome are measured in non-overlapping datasets, allow for minimal false-positive rates and increased sample sizes. In the MR analysis, random-effects inverse variance weighting (IVW) was used as the primary method, and MR-Egger regression, weighted median models, simple mode, and weighted mode were used as secondary methods. The walk ratio test was used when <3 SNPs were available for the analysis. To prevent potential violations of MR assumptions, we tested for pleiotropy (through MR-Egger regression intercepts, and MR Pleiotropy Residual Sum and Outlier (MR-PRESSO) global test) and heterogeneity (based on Cochrane Q values).  In two-sample MR, we first performed preliminary analyses based on IVW and four other secondary analyses. In the sensitivity analysis, to avoid reverse causation, the Steiger test was conducted for each SNP to determine whether the exposure R2 (the variance of the disease/trait explained by the selected SNP) was greater than the outcome R2, excluding SNPs that tested “False” (outcome R2 > exposure R2). When pleiotropy or heterogeneity was identified (P < 0.05), then we applied MRPRESSO to identify and eliminate outliers until no more outliers are identified. We then performed Radial-MR to determine whether outliers were present (the threshold was set at 0.05), and if outliers were present, they were eliminated and Radial-MR was repeated until no outliers were present. After excluding the above SNPs, the analyses were reconducted. |
|  | a) | Describe how quantitative variables were handled in the analyses (i.e., scale, units, model) | 5 | The daytime napping category (never, sometimes, or usually) was considered a continuous variable in the MR analysis. |
|  | b) | Describe how genetic variants were handled in the analyses and, if applicable, how their weights were selected | 5-6 | To ensure the reliability of the findings, the PLINK clumping method with a stringent clumping threshold (r2 < 0.001, LD distance = 10,000 kb) was applied. This ensured that SNPs in the residual linkage-disequilibrium (LD) within a particular window were pruned to evaluate the bias caused by the residual LD of genetic variants. For harmonization, the strand for non-palindromic SNPs was corrected. For palindromic SNPs, rather than excluding them from the analysis, an attempt was made to infer the alleles on the positive strand using allele frequencies. |
|  | c) | Describe the MR estimator (e.g. two-stage least squares, Wald ratio) and related statistics. Detail the included covariates and, in case of two-sample MR, whether the same covariate set was used for adjustment in the two samples |  | Used GWAS summary statistics |
|  | d) | Explain how missing data were addressed |  | Used GWAS summary statistics |
|  | e) | If applicable, indicate how multiple testing was addressed |  | Does not apply |
| 7 | **Assessment of assumptions** | Describe any methods or prior knowledge used to assess the assumptions or justify their validity | 6 | To prevent potential violations of MR assumptions, we tested for pleiotropy (through MR-Egger regression intercepts, and MR Pleiotropy Residual Sum and Outlier (MR-PRESSO) global test) and heterogeneity (based on Cochrane Q values). |
| 8 | **Sensitivity analyses and additional analyses** | Describe any sensitivity analyses or additional analyses performed (e.g. comparison of effect estimates from different approaches, independent replication, bias analytic techniques, validation of instruments, simulations) | 7 | In the sensitivity analysis, to avoid reverse causation, the Steiger test was conducted for each SNP to determine whether the exposure R2 (the variance of the disease/trait explained by the selected SNP) was greater than the outcome R2, excluding SNPs that tested “False” (outcome R2 > exposure R2). When pleiotropy or heterogeneity was identified (P < 0.05), then we applied MRPRESSO to identify and eliminate outliers until no more outliers are identified. We then performed Radial-MR to determine whether outliers were present (the threshold was set at 0.05), and if outliers were present, they were eliminated and Radial-MR was repeated until no outliers were present. After excluding the above SNPs, the analyses were reconducted. |
| 9 | **Software and pre-registration** |  |  |  |
|  | a) | Name statistical software and package(s), including version and settings used | 7 | Statistical analysis was conducted in R, Version 4.2.3. The MR analysis was conducted using the “TwoSampleMR” (Version 0.5.6) and “RadialMR” software package. |
|  | b) | State whether the study protocol and details were pre-registered (as well as when and where) |  | Does not apply |
|  | **RESULTS** |  |  |  |
| 10 | **Descriptive data** |  |  |  |
|  | a) | Report the numbers of individuals at each stage of included studies and reasons for exclusion. Consider use of a flow diagram |  | Does not apply |
|  | b) | Report summary statistics for phenotypic exposure(s), outcome(s), and other relevant variables (e.g. means, SDs, proportions) | 5 | For determining the daytime napping frequency, 104 independent single nucleotide polymorphisms (SNPs) (P < 5×10−8, r2 < 0.001, distance = 10,000 kb) from the GWAS study at the UK Biobank (n = 452,633) were used. These SNPs were replicated and validated in the 23andMe cohort (n = 541,333). The daytime napping category (never, sometimes, or usually) was considered a continuous variable in the MR analysis. Genotyping, quality control, and interpolation procedures for the UK Biobank data have been described elsewhere.  The GWAS summary data for PD were obtained from GWAS Catalog (https://www.ebi.ac.uk/gwas/home) and included 42,792 PD patients and 568,693 controls (Study ID: GCST90275127). |
|  | c) | If the data sources include meta-analyses of previous studies, provide the assessments of heterogeneity across these studies |  | Does not apply |
|  | d) | For two-sample MR:  i.  Provide justification of the similarity of the genetic variant-exposure associations between the exposure and outcome samples  ii.  Provide information on the number of individuals who overlap between the exposure and outcome studies |  | Does not apply  No overlap. |
| 11 | **Main results** |  |  |  |
|  | a) | Report the associations between genetic variant and exposure, and between genetic variant and outcome, preferably on an interpretable scale |  | Does not apply |
|  | b) | Report MR estimates of the relationship between exposure and outcome, and the measures of uncertainty from the MR analysis, on an interpretable scale, such as odds ratio or relative risk per SD difference | 8 | Table S14 in Additional file 1 present the two-sample MR results before excluding outliers identified by MRPRESSO or Radial-MR. The F-statistics for individual SNPs presented in Table S15 in Additional file 1. Regarding the impact of daytime napping on the incidence of PD, the random-effects IVW model shows no significant association between them (OR, 0.816, 95%CI, 0.510 to 1.304). This result remained consistent in other models. No heterogeneity or horizontal pleiotropy was found (P > 0.05). When PD as exposure, the random-effects IVW model showed no significant association between genetic liability to PD and daytime napping (β, 0.008, 95%CI, -0.006 to 0.022). Besides, IVW and MR-Egger revealed the presence of heterogeneity and MRPRESSO global test showed the presence of horizontal pleiotropy (P < 0.05). In the sensitivity analysis, SNPs that did not pass the Steiger test, and outliers identified by MRPRESSO or Radial-MR were displayed in Table S16 in Additional file 1. After the outliers were removed, no significant heterogeneity or horizontal pleiotropy was observed (Additional file 1: Table S17). The random-effects IVW indicated that no significant association between genetic liability to PD and daytime napping (β, -0.003; 95% CI, - 0.008 to 0.002). This result remained consistent in other models. Moreover, the leave-one-out method was used to test the stability of the results (Additional file 2: Fig.S2-S3). |
|  | c) | If relevant, consider translating estimates of relative risk into absolute risk for a meaningful time period |  | Does not apply |
|  | d) | Consider plots to visualize results (e.g. forest plot, scatterplot of associations between genetic variants and outcome versus between genetic variants and exposure) |  | Additional file 2: Fig.S2-S3 |
| 12 | **Assessment of assumptions** |  |  |  |
|  | a) | Report the assessment of the validity of the assumptions |  | Table S14,15,17 in Additional file 1 |
|  | b) | Report any additional statistics (e.g., assessments of heterogeneity across genetic variants, such as *I^2^*, Q statistic or E-value) |  | Table S14,15,17 in Additional file 1 |
| 13 | **Sensitivity analyses and additional analyses** |  |  |  |
|  | a) | Report any sensitivity analyses to assess the robustness of the main results to violations of the assumptions | 8 | In the sensitivity analysis, SNPs that did not pass the Steiger test, and outliers identified by MRPRESSO or Radial-MR were displayed in Table S16 in Additional file 1. After the outliers were removed, no significant heterogeneity or horizontal pleiotropy was observed (Additional file 1: Table S17). The random-effects IVW indicated that no significant association between genetic liability to PD and daytime napping (β, -0.003; 95% CI, - 0.008 to 0.002). This result remained consistent in other models. Moreover, the leave-one-out method was used to test the stability of the results (Additional file 2: Fig.S2-S3). |
|  | b) | Report results from other sensitivity analyses or additional analyses | 8 | After the outliers were removed, no significant heterogeneity or horizontal pleiotropy was observed (Additional file 1: Table S17). The random-effects IVW indicated that no significant association between genetic liability to PD and daytime napping (β, -0.003; 95% CI, - 0.008 to 0.002). This result remained consistent in other models. Moreover, the leave-one-out method was used to test the stability of the results (Additional file 2: Fig.S2-S3). |
|  | c) | Report any assessment of direction of causal relationship (e.g., bidirectional MR) |  | Table S14,16,17 in Additional file 1 |
|  | d) | When relevant, report and compare with estimates from non-MR analyses |  | Presented in the Results section. |
|  | e) | Consider additional plots to visualize results (e.g., leave-one-out analyses) |  | Additional file 2: Fig.S2-S3 |
|  | **DISCUSSION** |  |  |  |
| 14 | **Key results** | Summarize key results with reference to study objectives | 9 | This large prospective cohort study found that the higher frequency and longer duration of daytime napping were associated with an increased PD risk. The MR analysis revealed no reciprocal causal relationship between the daytime napping frequency and the PD risk. Additionally, the higher frequency of daytime napping was associated with an increase in the immune inflammatory response, which elevated the PD risk. Moreover, no significant interaction was observed between the frequency and duration of daytime napping and PRS in their effect on the PD risk. |
| 15 | **Limitations** | Discuss limitations of the study, taking into account the validity of the IV assumptions, other sources of potential bias, and imprecision. Discuss both direction and magnitude of any potential bias and any efforts to address them | 10 | Third, a fundamental assumption of MR is that SNPs are not linked to any confounders of the exposure or the outcome. Even when pleiotropic bias is taken into account, no MR study can entirely eliminate the possibility of pleiotropic bias. |
| 16 | **Interpretation** |  |  |  |
|  | a) | Meaning: Give a cautious overall interpretation of results in the context of their limitations and in comparison with other studies | 9 | The observational study results align with those of numerous epidemiologic studies, indicating that the increased daytime napping frequency and longer nap duration are linked to a higher PD risk. In a multicenter prospective study, 2920 men without a history of PD were followed for 11 years. The study ultimately revealed 106 PD events and demonstrated a correlation between objectively prolonged napping and a higher PD risk in older men[6]. A multicenter cohort study of 2,675 community-dwelling older women reported that both subjective and objective naps were associated with PD[7]. In a United States-based cohort study with 213,885 volunteers having no history of PD, subjective daytime napping was associated with a higher PD risk[8]. A previous study based on the Honolulu-Asia Aging Study revealed that subjective daytime napping is associated with the PD risk in men[40].  The MR analysis revealed no causal relationship between the daytime napping frequency and the PD risk. Nevertheless, caution is warranted in interpreting these findings because of the small sample size and low precision of our study. The analyses were also constrained by a rudimentary assessment of the daytime napping frequency through a questionnaire, which resulted in the lack of details on duration or timing[26]. Our attempts to partially validate the specificity of identified loci, transitioning from self-reporting of data to objective determination through accelerometers, may have faced limitations because of the phenotypic differences between self-report and accelerometer data. Additionally, the relatively small sample size in the accelerometer subsample and the time lag between measurements, with accelerometers worn between 2 and 10 years after the study baseline constrained the assessment. Moreover, additional larger studies using GWAS-based cohorts and MR approaches are necessary for thoroughly investigating the relationship between the daytime napping frequency and the PD risk. |
|  | b) | Mechanism: Discuss underlying biological mechanisms that could drive a potential causal relationship between the investigated exposure and the outcome, and whether the gene-environment equivalence assumption is reasonable. Use causal language carefully, clarifying that IV estimates may provide causal effects only under certain assumptions | 9-11 | Presented in the paragraph 4-6 of discussion section, and limitation section. |
|  | c) | Clinical relevance: Discuss whether the results have clinical or public policy relevance, and to what extent they inform effect sizes of possible interventions | 10 | The direction of the relationship is a key issue in the study of sleep and PD. Thus, benefiting from the long follow-up of the UK Biobank study, we found similar results when PD cases were excluded at risk 2 years and 4 years after the baseline, a method reported in previous studies[6]. |
| 17 | **Generalizability** | Discuss the generalizability of the study results (a) to other populations, (b) across other exposure periods/timings, and (c) across other levels of exposure | 11 | Finally, the UK Biobank participants were predominantly Europeans. Further studies are warranted to investigate the extent to which these findings can be applied to other populations. |
|  | **OTHER INFORMATION** |  |  |  |
| 18 | **Funding** | Describe sources of funding and the role of funders in the present study and, if applicable, sources of funding for the databases and original study or studies on which the present study is based | 11 | Presented in Funding section. |
| 19 | **Data and data sharing** | Provide the data used to perform all analyses or report where and how the data can be accessed, and reference these sources in the article. Provide the statistical code needed to reproduce the results in the article, or report whether the code is publicly accessible and if so, where |  | Presented in Availability of Data and Materials statement. |
| 20 | **Conflicts of Interest** | All authors should declare all potential conflicts of interest |  | The authors declare that they have no competing interests. |

This checklist is copyrighted by the Equator Network under the Creative Commons Attribution 3.0 Unported (CC BY 3.0) license.

1. Skrivankova VW, Richmond RC, Woolf BAR, Yarmolinsky J, Davies NM, Swanson SA, et al. Strengthening the Reporting of Observational Studies in Epidemiology using Mendelian Randomization (STROBE-MR) Statement. JAMA. 2021;under review.

2. Skrivankova VW, Richmond RC, Woolf BAR, Davies NM, Swanson SA, VanderWeele TJ, et al. Strengthening the Reporting of Observational Studies in Epidemiology using Mendelian Randomisation (STROBE-MR): Explanation and Elaboration. BMJ. 2021;375:n2233.
