# Supplementary material for: Daytime napping and the incidence of Parkinson’s disease: a prospective cohort study with Mendelian randomization
Source: BMC Med. 2024 Aug 13;22:326. doi: 10.1186/s12916-024-03497-7 (PMC11321229; doi:10.1186/s12916-024-03497-7)
Supplement: Supplementary file 2 — Additional file 2: Text S1, Table S1-S15, and Figures S1, S3. Text S1- [Assessment of covariates]. Table S1- [Baseline sleep characteristics of participants based on frequency of daytime naps]. Table S2- [Baseline characteristics of participants based on daytime napping during]. Table S3- [Baseline characteristics of participants based on daytime napping frequency (First repeat assessment visit (2012–2013))]. Table S4- [Baseline characteristics of participants based on daytime napping frequency (Imaging visit (2014 +))]. Table S5- [Baseline characteristics of participants based on daytime napping frequency (First repeat imaging visit (2019 +))]. Table S6- [Daytime nap frequency and Parkinson’s disease prevalence when using different time points as a baseline]. Table S7- [Relationship between daytime napping duration and onset of Parkinson’s disease when napping was taking place at different times (N = 78,141)]. Table S8- [Association between daytime napping and incident Parkinson’s disease after excluding participants who experienced an outcome event within the first two years of follow-up]. Table S9- [Association between daytime napping and incident Parkinson’s disease after excluding participants who experienced an outcome event within the first four years of follow-up]. Table S10- [Association between daytime napping and incident Parkinson’s disease after excluding participants who self-reported Parkinson’s disease]. Table S11- [Association between daytime napping and incident Parkinson’s disease after excluding participants who worked night shifts]. Table S12- [Association between daytime napping and incident Parkinson’s disease after excluding participants with sleep disorders]. Table S13- [Associations between daytime napping and incident Parkinson’s disease by treating all-cause death as a competing risk]. Table S14- [Analysis of the association between daytime napping and PD with bidirectional two-sample Mendelian randomization]. Table S15- [Outliers identif [file 12916_2024_3497_MOESM2_ESM.docx]

**ADDITIONAL FILE 2**

**Text. S1**. Assessment of covariates

The covariates preliminarily included in this study included age, body mass index (kg/m^2^), gender (Male/ Female), ethnicity (White/ Black/ Asian/ Others), education level (College or University degree/Vocational/Any school degree/Others), household income (Greater than 100,000/ 18,000 to 100,000/ Less than 18,000/ Unknown), Current tobacco smoking (Yes, on most or all days/ Only occasionally/ No), drinking status (Current/ Previous/ Never), physical activity (minutes per week), sleep duration (hours per day) and family history of Parkinson's disease (Yes/ No).

The sleep-related covariates that were further included were: difficulty in getting up in the morning (Very easy/ Fairly easy/ Not very easy/ Not at all easy), morning evening person chronotype (Definitely a 'morning' person/ Definitely an 'evening' person/ More a 'morning' than 'evening' person/ More an 'evening' than a 'morning' person), insomnia (Usually/Sometimes/ Never/rarely), snoring (Yes/ No), and daytime dozing (All of the time/Often/Sometimes/Never/rarely).

Body mass index constructed from height and weight measured during the initial Assessment Centre visit. other covariates were obtained from baseline self-report or interview.

**Table S1** Baseline sleep characteristics of participants based on frequency of daytime naps.

| Characteristics | | Daytime napping | | | | *P-*value |
| --- | --- | --- | --- | --- | --- | --- |
|  |  | Overall | Never/rarely | Sometimes | Usually |  |
| Participants, n |  | 393302 | 224646 | 148094 | 20562 |  |
| Getting up in morning | Do not know | 197 (0.1) | 79 (0.0) | 79 (0.1) | 39 (0.2) | <0.001 |
|  | Fairly easy | 196433 (49.9) | 114556 (51.0) | 73229 (49.4) | 8648 (42.1) |  |
|  | Not at all easy | 14440 (3.7) | 7167 (3.2) | 5888 (4.0) | 1385 (6.7) |  |
|  | Not very easy | 54116 (13.8) | 30935 (13.8) | 20412 (13.8) | 2769 (13.5) |  |
|  | Prefer not to answer | 53 (0.0) | 15 (0.0) | 28 (0.0) | 10 (0.0) |  |
|  | Very easy | 128063 (32.6) | 71894 (32.0) | 48458 (32.7) | 7711 (37.5) |  |
| Morning evening person chronotype | Definitely a 'morning' person | 96057 (24.4) | 51924 (23.1) | 37918 (25.6) | 6215 (30.2) | <0.001 |
|  | Definitely an 'evening' person | 32202 (8.2) | 18150 (8.1) | 11906 (8.0) | 2146 (10.4) |  |
|  | Do not know | 36328 (9.2) | 21411 (9.5) | 13078 (8.8) | 1839 (8.9) |  |
|  | More a 'morning' than 'evening' person | 127724 (32.5) | 74008 (32.9) | 47925 (32.4) | 5791 (28.2) |  |
|  | More an 'evening' than a 'morning' person | 100381 (25.5) | 58840 (26.2) | 37001 (25.0) | 4540 (22.1) |  |
|  | Prefer not to answer | 610 (0.2) | 313 (0.1) | 266 (0.2) | 31 (0.2) |  |
| Sleeplessness insomnia | Never/rarely | 99279 (25.2) | 61065 (27.2) | 33035 (22.3) | 5179 (25.2) | <0.001 |
|  | Prefer not to answer | 153 (0.0) | 64 (0.0) | 69 (0.0) | 20 (0.1) |  |
|  | Sometimes | 186639 (47.5) | 105517 (47.0) | 73220 (49.4) | 7902 (38.4) |  |
|  | Usually, | 107231 (27.3) | 58000 (25.8) | 41770 (28.2) | 7461 (36.3) |  |
| Snoring | Do not know | 20856 (5.3) | 11309 (5.0) | 8279 (5.6) | 1268 (6.2) | <0.001 |
|  | No | 231106 (58.8) | 138802 (61.8) | 81584 (55.1) | 10720 (52.1) |  |
|  | Prefer not to answer | 3811 (1.0) | 1917 (0.9) | 1662 (1.1) | 232 (1.1) |  |
|  | Yes | 137529 (35.0) | 72618 (32.3) | 56569 (38.2) | 8342 (40.6) |  |
| Daytime dozing sleeping | All of the time | 20 (0.0) | 4 (0.0) | 7 (0.0) | 9 (0.0) | <0.001 |
|  | Do not know | 799 (0.2) | 239 (0.1) | 455 (0.3) | 105 (0.5) |  |
|  | Never/rarely | 301589 (76.7) | 198961 (88.6) | 91816 (62.0) | 10812 (52.6) |  |
|  | Often | 10244 (2.6) | 1226 (0.5) | 5513 (3.7) | 3505 (17.0) |  |
|  | Prefer not to answer | 160 (0.0) | 40 (0.0) | 101 (0.1) | 19 (0.1) |  |
|  | Sometimes | 80490 (20.5) | 24176 (10.8) | 50202 (33.9) | 6112 (29.7) |  |

Data are presented as n (%).

The χ2 tests were used for categorical variables.

**Table S2** Baseline characteristics of participants based on daytime napping during.

| Characteristics | | Daytime napping | | | | *P-*value |
| --- | --- | --- | --- | --- | --- | --- |
|  |  | Overall | 0 | <1 hour | ≥1 hour |  |
| Participants, n | | 78141 | 20837 | 43914 | 13390 |  |
| Age, Mean(SD) | | 55.96 (7.83) | 55.74 (7.88) | 55.92 (7.82) | 56.41 (7.79) | <0.001 |
| Sex, n (%) | Female | 42874 (54.9) | 11553 (55.4) | 24592 (56.0) | 6729 (50.3) | <0.001 |
|  | Male | 35267 (45.1) | 9284 (44.6) | 19322 (44.0) | 6661 (49.7) |  |
| Ethnicity, n (%) | White | 73146 (93.6) | 19644 (94.3) | 41195 (93.8) | 12307 (91.9) | <0.001 |
|  | Black | 195 (0.2) | 36 (0.2) | 99 (0.2) | 60 (0.4) |  |
|  | Asia | 736 (0.9) | 154 (0.7) | 374 (0.9) | 208 (1.6) |  |
|  | Other | 4064 (5.2) | 1003 (4.8) | 2246 (5.1) | 815 (6.1) |  |
| BMI (kg/m2), Mean(SD) | | 26.60 (4.43) | 25.93 (4.08) | 26.61 (4.36) | 27.62 (4.96) | <0.001 |
| Household income, n (%) | Greater than 100,000 | 5727 (7.3) | 1795 (8.6) | 3199 (7.3) | 733 (5.5) | <0.001 |
|  | 18,000 to 100,000 | 56581 (72.4) | 15364 (73.7) | 32010 (72.9) | 9207 (68.8) |  |
|  | Less than 18,000 | 9718 (12.4) | 2083 (10.0) | 5263 (12.0) | 2372 (17.7) |  |
|  | Unknow | 6115 (7.8) | 1595 (7.7) | 3442 (7.8) | 1078 (8.1) |  |
| Education, n (%) | Any school degree | 10480 (13.4) | 2869 (13.8) | 5834 (13.3) | 1777 (13.3) | <0.001 |
|  | College or University degree | 35619 (45.6) | 10306 (49.5) | 20010 (45.6) | 5303 (39.6) |  |
|  | Vocational | 2909 (3.7) | 648 (3.1) | 1692 (3.9) | 569 (4.2) |  |
|  | Other | 29133 (37.3) | 7014 (33.7) | 16378 (37.3) | 5741 (42.9) |  |
| Current tobacco smoking, n (%) | No | 72994 (93.4) | 19884 (95.4) | 41186 (93.8) | 11924 (89.1) | <0.001 |
|  | Only occasionally | 1758 (2.2) | 389 (1.9) | 975 (2.2) | 394 (2.9) |  |
|  | Yes, on most or all days | 3389 (4.3) | 564 (2.7) | 1753 (4.0) | 1072 (8.0) |  |
| Alcohol, n (%) | Never | 2064 (2.6) | 514 (2.5) | 1123 (2.6) | 427 (3.2) | <0.001 |
|  | Previous | 2037 (2.6) | 457 (2.2) | 1099 (2.5) | 481 (3.6) |  |
|  | Current | 74040 (94.8) | 19866 (95.3) | 41692 (94.9) | 12482 (93.2) |  |
| Physical activity (minutes/week), Mean(SD) | | 2522.49 (2457.36) | 2588.71 (2420.44) | 2508.75 (2439.27) | 2464.49 (2568.99) | <0.001 |
| Sleep duration (hours/day), Mean(SD) | | 7.17 (0.96) | 7.09 (0.89) | 7.18 (0.95) | 7.27 (1.11) | <0.001 |
| Family history of PD, n (%) | No | 74557 (95.4) | 19859 (95.3) | 41928 (95.5) | 12770 (95.4) | 0.602 |
|  | Yes | 3584 (4.6) | 978 (4.7) | 1986 (4.5) | 620 (4.6) |  |

Abbreviation: BMI, body mass index; PD, Parkinson’s disease; SD, standard deviation.

Data are presented as mean (standard deviation) or n (%). The one-way ANOVA tests were used for continuous variables and χ2 tests were used for categorical variables.

**Table S3** Baseline characteristics of participants based on daytime napping frequency (First repeat assessment visit (2012–2013)).

| Characteristics | | Daytime napping | | | | *P-*value |
| --- | --- | --- | --- | --- | --- | --- |
|  |  | Overall | Never/rarely | Sometimes | Usually, |  |
| Participants, n | | 16535 | 9536 | 6055 | 944 |  |
| Age, Mean(SD) |  | 56.98 (7.43) | 55.96 (7.46) | 58.07 (7.18) | 60.35 (6.72) | <0.001 |
| Sex, n (%) | Female | 8103 (49.0) | 5363 (56.2) | 2483 (41.0) | 257 (27.2) | <0.001 |
|  | Male | 8432 (51.0) | 4173 (43.8) | 3572 (59.0) | 687 (72.8) | |
| Ethnicity, n (%) | White | 15769 (95.4) | 9075 (95.2) | 5801 (95.8) | 893 (94.6) | 0.016 |
|  | Black | 33 (0.2) | 12 (0.1) | 19 (0.3) | 2 (0.2) |  |
|  | Asia | 126 (0.8) | 72 (0.8) | 43 (0.7) | 11 (1.2) |  |
|  | Other | 607 (3.7) | 377 (4.0) | 192 (3.2) | 38 (4.0) |  |
| BMI (kg/m2), Mean(SD) |  | 26.83 (4.46) | 26.39 (4.27) | 27.35 (4.58) | 27.96 (4.93) | <0.001 |
| Household income, n (%) | Greater than 100,000 | 805 (4.9) | 554 (5.8) | 228 (3.8) | 23 (2.4) | <0.001 |
|  | 18,000 to 100,000 | 12036 (72.8) | 7034 (73.8) | 4360 (72.0) | 642 (68.0) | |
|  | Less than 18,000 | 2452 (14.8) | 1240 (13.0) | 1011 (16.7) | 201 (21.3) | |
|  | Unknow | 1242 (7.5) | 708 (7.4) | 456 (7.5) | 78 (8.3) |  |
| Education, n (%) | Any school degree | 2089 (12.6) | 1233 (12.9) | 741 (12.2) | 115 (12.2) | <0.001 |
|  | College or University degree | 7577 (45.8) | 4564 (47.9) | 2629 (43.4) | 384 (40.7) | |
|  | Vocational | 537 (3.2) | 298 (3.1) | 211 (3.5) | 28 (3.0) |  |
|  | Other | 6332 (38.3) | 3441 (36.1) | 2474 (40.9) | 417 (44.2) | |
| Current tobacco smoking, n (%) | No | 15490 (93.7) | 8988 (94.3) | 5647 (93.3) | 855 (90.6) | <0.001 |
|  | Only occasionally | 340 (2.1) | 197 (2.1) | 118 (1.9) | 25 (2.6) |  |
|  | Yes, on most or all days | 705 (4.3) | 351 (3.7) | 290 (4.8) | 64 (6.8) |  |
| Alcohol, n (%) | Never | 479 (2.9) | 270 (2.8) | 176 (2.9) | 33 (3.5) | <0.001 |
|  | Previous | 399 (2.4) | 184 (1.9) | 175 (2.9) | 40 (4.2) |  |
|  | Current | 15657 (94.7) | 9082 (95.2) | 5704 (94.2) | 871 (92.3) | |
| Physical activity (minutes/week), Mean(SD) | | 2457.65 (2458.50) | 2443.59 (2410.17) | 2461.10 (2502.89) | 2577.65 (2646.89) | 0.276 |
| Sleep duration (hours/day), Mean(SD) | | 7.21 (1.00) | 7.14 (0.94) | 7.26 (1.03) | 7.58 (1.21) | <0.001 |
| Family history of PD, n (%) | No | 15669 (94.8) | 9043 (94.8) | 5734 (94.7) | 892 (94.5) | 0.871 |
|  | Yes | 866 (5.2) | 493 (5.2) | 321 (5.3) | 52 (5.5) |  |

Abbreviation: BMI, body mass index; PD, Parkinson’s disease; SD, standard deviation.

Data are presented as mean (standard deviation) or n (%). The one-way ANOVA tests were used for continuous variables and χ2 tests were used for categorical variables.

**Table S4** Baseline characteristics of participants based on daytime napping frequency (Imaging visit (2014+)).

| Characteristics | | Daytime napping | | | | *P-*value |
| --- | --- | --- | --- | --- | --- | --- |
|  |  | Overall | Never/rarely | Sometimes | Usually, |  |
| Participants, n | | 54769 | 30849 | 20540 | 3380 |  |
| Age, Mean(SD) |  | 54.69 (7.56) | 53.72 (7.43) | 55.60 (7.51) | 57.98 (7.44) | <0.001 |
| Sex, n (%) | Female | 27404 (50.0) | 17615 (57.1) | 8886 (43.3) | 903 (26.7) | <0.001 |
|  | Male | 27365 (50.0) | 13234 (42.9) | 11654 (56.7) | 2477 (73.3) | |
| Ethnicity, n (%) | White | 51351 (93.8) | 28922 (93.8) | 19276 (93.8) | 3153 (93.3) | <0.001 |
|  | Black | 143 (0.3) | 56 (0.2) | 75 (0.4) | 12 (0.4) |  |
|  | Asia | 647 (1.2) | 350 (1.1) | 239 (1.2) | 58 (1.7) |  |
|  | Other | 2628 (4.8) | 1521 (4.9) | 950 (4.6) | 157 (4.6) | |
| BMI (kg/m2), Mean (SD) |  | 26.62 (4.25) | 26.20 (4.11) | 27.13 (4.34) | 27.42 (4.43) | <0.001 |
| Household income, n (%) | Greater than 100,000 | 4299 (7.8) | 2712 (8.8) | 1391 (6.8) | 196 (5.8) | <0.001 |
|  | 18,000 to 100,000 | 41118 (75.1) | 23230 (75.3) | 15386 (74.9) | 2502 (74.0) | |
|  | Less than 18,000 | 5529 (10.1) | 2687 (8.7) | 2360 (11.5) | 482 (14.3) | |
|  | Unknow | 3823 (7.0) | 2220 (7.2) | 1403 (6.8) | 200 (5.9) | |
| Education, n (%) | Any school degree | 7186 (13.1) | 4217 (13.7) | 2600 (12.7) | 369 (10.9) | <0.001 |
|  | College or University degree | 26035 (47.5) | 14898 (48.3) | 9468 (46.1) | 1669 (49.4) | |
|  | Vocational | 2246 (4.1) | 1286 (4.2) | 858 (4.2) | 102 (3.0) | |
|  | Other | 19302 (35.2) | 10448 (33.9) | 7614 (37.1) | 1240 (36.7) | |
| Current tobacco smoking, n (%) | No | 51294 (93.7) | 29070 (94.2) | 19090 (92.9) | 3134 (92.7) | <0.001 |
|  | Only occasionally | 1332 (2.4) | 697 (2.3) | 559 (2.7) | 76 (2.2) |  |
|  | Yes, on most or all days | 2143 (3.9) | 1082 (3.5) | 891 (4.3) | 170 (5.0) | |
| Alcohol, n (%) | Never | 1309 (2.4) | 748 (2.4) | 468 (2.3) | 93 (2.8) | <0.001 |
|  | Previous | 1190 (2.2) | 560 (1.8) | 516 (2.5) | 114 (3.4) | |
|  | Current | 52270 (95.4) | 29541 (95.8) | 19556 (95.2) | 3173 (93.9) | |
| Physical activity (minutes/week), Mean (SD) | | 2472.34 (2444.19) | 2457.91 (2389.20) | 2487.90 (2504.53) | 2509.38 (2566.00) | 0.261 |
| Sleep duration (hours/day), Mean (SD) | | 7.17 (0.96) | 7.11 (0.92) | 7.20 (0.98) | 7.44 (1.11) | <0.001 |
| Family history of PD, n (%) | No | 52065 (95.1) | 29384 (95.3) | 19479 (94.8) | 3202 (94.7) | 0.068 |
|  | Yes | 2704 (4.9) | 1465 (4.7) | 1061 (5.2) | 178 (5.3) | |

Abbreviation: BMI, body mass index; PD, Parkinson’s disease; SD, standard deviation.

Data are presented as mean (standard deviation) or n (%). The one-way ANOVA tests were used for continuous variables and χ2 tests were used for categorical variables.

**Table S5** Baseline characteristics of participants based on daytime napping frequency (First repeat imaging visit (2019+)).

| Characteristics | | Daytime napping | | | | *P-*value |
| --- | --- | --- | --- | --- | --- | --- |
|  |  | Overall | Never/rarely | Sometimes | Usually, |  |
| Participants, n | | 4581 | 2665 | 1649 | 267 |  |
| Age, Mean(SD) |  | 52.88 (7.42) | 51.95 (7.17) | 53.88 (7.60) | 56.03 (7.12) | <0.001 |
| Sex, n (%) | Female | 2286 (49.9) | 1533 (57.5) | 680 (41.2) | 73 (27.3) | <0.001 |
|  | Male | 2295 (50.1) | 1132 (42.5) | 969 (58.8) | 194 (72.7) | |
| Ethnicity, n (%) | White | 4338 (94.7) | 2526 (94.8) | 1563 (94.8) | 249 (93.3) | 0.271 |
|  | Black | 14 (0.3) | 5 (0.2) | 9 (0.5) | 0 (0.0) |  |
|  | Asia | 52 (1.1) | 30 (1.1) | 17 (1.0) | 5 (1.9) |  |
|  | Other | 177 (3.9) | 104 (3.9) | 60 (3.6) | 13 (4.9) |  |
| BMI (kg/m2), Mean(SD) |  | 26.39 (4.08) | 25.99 (3.88) | 26.94 (4.35) | 26.93 (3.76) | <0.001 |
| Household income, n (%) | Greater than 100,000 | 389 (8.5) | 252 (9.5) | 122 (7.4) | 15 (5.6) | 0.001 |
|  | 18,000 to 100,000 | 3536 (77.2) | 2054 (77.1) | 1283 (77.8) | 199 (74.5) | |
|  | Less than 18,000 | 402 (8.8) | 206 (7.7) | 159 (9.6) | 37 (13.9) | |
|  | Unknow | 254 (5.5) | 153 (5.7) | 85 (5.2) | 16 (6.0) |  |
| Education, n (%) | Any school degree | 655 (14.3) | 396 (14.9) | 219 (13.3) | 40 (15.0) | 0.031 |
|  | College or University degree | 2154 (47.0) | 1272 (47.7) | 758 (46.0) | 124 (46.4) | |
|  | Vocational | 202 (4.4) | 128 (4.8) | 70 (4.2) | 4 (1.5) |  |
|  | Other | 1570 (34.3) | 869 (32.6) | 602 (36.5) | 99 (37.1) | |
| Current tobacco smoking, n (%) | No | 4337 (94.7) | 2526 (94.8) | 1561 (94.7) | 250 (93.6) | 0.92 |
|  | Only occasionally | 95 (2.1) | 53 (2.0) | 36 (2.2) | 6 (2.2) |  |
|  | Yes, on most or all days | 149 (3.3) | 86 (3.2) | 52 (3.2) | 11 (4.1) |  |
| Alcohol, n (%) | Never | 98 (2.1) | 61 (2.3) | 33 (2.0) | 4 (1.5) | 0.269 |
|  | Previous | 80 (1.7) | 39 (1.5) | 33 (2.0) | 8 (3.0) |  |
|  | Current | 4403 (96.1) | 2565 (96.2) | 1583 (96.0) | 255 (95.5) | |
| Physical activity (minutes/week), Mean(SD) | | 2504.60 (2436.06) | 2534.19 (2443.40) | 2467.53 (2439.26) | 2438.08 (2345.73) | 0.615 |
| Sleep duration (hours/day), Mean(SD) | | 7.16 (0.92) | 7.12 (0.87) | 7.19 (0.93) | 7.43 (1.18) | <0.001 |
| Family history of PD, n (%) | No | 4339 (94.7) | 2523 (94.7) | 1561 (94.7) | 255 (95.5) | 0.839 |
|  | Yes | 242 (5.3) | 142 (5.3) | 88 (5.3) | 12 (4.5) |  |

Abbreviation: BMI, body mass index; PD, Parkinson’s disease; SD, standard deviation.

Data are presented as mean (standard deviation) or n (%). The one-way ANOVA tests were used for continuous variables and χ2 tests were used for categorical variables.

**Table S6** Daytime nap frequency and Parkinson's disease prevalence when using different time points as a baseline

| Time point | Daytime napping | HR (95%CI) *P*-value |
| --- | --- | --- |
| First repeat assessment visit (2012–2013) | Never/rarely | Ref |
|  | Sometimes | **2.22(1.30-3.80) 0.004** |
|  | Usually | **3.42(1.66-7.07) 0.001** |
| Imaging visit (2014+) | Never/rarely | Ref |
|  | Sometimes | **3.04(1.80-5.12) <0.001** |
|  | Usually | **4.18(2.11-8.27) <0.001** |
| First repeat imaging visit (2019+) | Never/rarely | Ref |
|  | Sometimes | 0(0.00-Inf) 0.999 |
|  | Usually | **8.58(1.19-62.03) 0.033** |

Abbreviation: HR, hazard ratio; BMI, body mass index.

Model adjusted for age, sex, race, household income, education, BMI, current tobacco smoking, physical activity, alcohol, sleep duration, and family history of Parkinson’s disease.

**Table S7** Relationship between daytime napping duration and onset of Parkinson's disease when napping was taking place at different times (N=78141).

| Time period for taking nap | | HR (95%CI) *P*-value |
| --- | --- | --- |
|  | 9:00am-12:00am | 1.00 (1.00-1.01) 0.107 |
|  | 13:00pm-15:00pm | **1.02 (1.01-1.03) <0.001** |
|  | 16:00pm-18:00pm | **1.01 (1.00-1.02) 0.001** |

Abbreviation: HR, hazard ratio; BMI, body mass index.

Model adjusted for age, sex, race, household income, education, BMI, current tobacco smoking, physical activity, alcohol, sleep duration, and family history of Parkinson’s disease.

**Table S8** Association between daytime napping and incident Parkinson’s disease after excluding participants who experienced an outcome event within the first two years of follow-up.

| Characteristic | | Sample size, n (%) | HR (95%CI) *P*-value |
| --- | --- | --- | --- |
| Frequency | Never/rarely | 224623 (57.1%) | 1.00(reference) |
|  | Sometimes | 148062 (37.7%) | 1.12 (1.02-1.23) 0.016 |
|  | Usually | 20554 (5.2%) | 1.32 (1.13-1.53) <0.001 |
| During | 0 hour | 20835 (26.7%) | 1.00(reference) |
|  | <1 hour | 43912 (56.2%) | 0.9(0.67-1.21) 0.477 |
|  | ≥1 hour | 13390 (17.1%) | 1.58(1.13-2.22) 0.007 |
|  | Continues | | 1.00 (1.00-1.01) <0.001 |

Abbreviation: HR, hazard ratio; BMI, body mass index.

Model adjusted for age, sex, race, household income, education, BMI, current tobacco smoking, physical activity, alcohol, sleep duration, and family history of Parkinson’s disease.

**Table S9** Association between daytime napping and incident Parkinson’s disease after excluding participants who experienced an outcome event within the first four years of follow-up.

| Characteristic | | Sample size, n (%) | HR (95%CI) *P*-value |
| --- | --- | --- | --- |
| Frequency | Never/rarely | 224566(57.1%) | 1.00(reference) |
|  | Sometimes | 148007(37.7%) | **1.13 (1.03-1.24) 0.013** |
|  | Usually | 20537(5.2%) | **1.30 (1.11-1.52) 0.001** |
| During | 0 hour | 20828(26.7%) | 1.00(reference) |
|  | <1 hour | 43904(56.2%) | 0.93(0.69-1.27) 0.656 |
|  | >=1 hour | 13385(17.1%) | **1.6(1.13-2.27) 0.008** |
|  | Continues | | **1.00 (1.00-1.01) <0.001** |

Abbreviation: HR, hazard ratio; BMI, body mass index.

Model adjusted for age, sex, race, household income, education, BMI, current tobacco smoking, physical activity, alcohol, sleep duration, and family history of Parkinson’s disease.

**Table S10** Association between daytime napping and incident Parkinson’s disease after excluding participants who self-reported Parkinson's disease.

| Characteristic | | Sample size, n (%) | HR (95%CI) *P*-value |
| --- | --- | --- | --- |
| Frequency | Never/rarely | 224608 (57.1%) | 1.00(reference) |
|  | Sometimes | 148064 (37.7%) | **1.13 (1.03-1.23) 0.009** |
|  | Usually | 20559 (5.2%) | **1.33 (1.15-1.55) <0.001** |
| During | 0 hour | 20833 (26.7%) | 1.00(reference) |
|  | ≥1 hour | 43907 (56.2%) | 0.88 (0.66-1.18) 0.391 |
|  | >=1 hour | 13385 (17.1%) | **1.54 (1.10-2.15) 0.011** |
|  | Continues | | **1.01 (1.00-1.01) <0.001** |

Abbreviation: HR, hazard ratio; BMI, body mass index.

Model adjusted for age, sex, race, household income, education, BMI, current tobacco smoking, physical activity, alcohol, sleep duration, and family history of Parkinson’s disease.

**Table S11** Association between daytime napping and incident Parkinson’s disease after excluding participants who worked night shifts.

| Characteristic | | Sample size, n(%) | HR (95%CI) *P*-value |
| --- | --- | --- | --- |
| Frequency | Never/rarely | 217697 (57.4%) | 1.00(reference) |
|  | Sometimes | 141863 (37.4%) | 1.12 (1.03-1.23) 0.011 |
|  | Usually | 19861 (5.2%) | 1.33 (1.14-1.55) <0.001 |
| During | 0 hour | 20395 (26.8%) | 1.00(reference) |
|  | <1 hour | 42788 (56.3%) | 0.90 (0.67-1.21) 0.494 |
|  | ≥1 hour | 12831 (16.9%) | 1.57 (1.12-2.20) 0.009 |
|  | Continues | | 1.00 (1.00-1.01) <0.001 |

Abbreviation: HR, hazard ratio; BMI, body mass index.

Model adjusted for age, sex, race, household income, education, BMI, current tobacco smoking, physical activity, alcohol, sleep duration, and family history of Parkinson’s disease.

**Table S12** Association between daytime napping and incident Parkinson’s disease after excluding participants with sleep disorders

| Characteristic | | Sample size, n(%) | HR (95%CI) *P*-value |
| --- | --- | --- | --- |
| Frequency | Never/rarely | 217557 (57.6%) | 1.00(reference) |
|  | Sometimes | 141025 (37.4%) | **1.13 (1.03-1.24) 0.011** |
|  | Usually | 18934 (5.0%) | **1.30 (1.11-1.53) 0.001** |
| During | 0 hour | 20307 (26.9%) | 1.00(reference) |
|  | <1 hour | 42466 (56.3%) | 0.93 (0.68-1.26) 0.628 |
|  | ≥1 hour | 12710 (16.8%) | **1.63 (1.15-2.31) 0.006** |
|  | Continues | | **1.00 (1.00-1.01) <0.001** |

Abbreviation: HR, hazard ratio; BMI, body mass index.

Model adjusted for age, sex, race, household income, education, BMI, current tobacco smoking, physical activity, alcohol, sleep duration, and family history of Parkinson’s disease.

**Table S13** Associations between daytime napping and incident Parkinson’s disease by treating all-cause death as a competing risk

| Characteristic |  | HR (95%CI) | *P*-value |
| --- | --- | --- | --- |
| Frequency | Never/rarely | 1.00(reference) |  |
|  | Sometimes | 1.05(0.94-1.17) | 0.410 |
|  | Usually | 1.22(1.01-1.48) | 0.037 |
| During | 0 hour | 1.00(reference) |  |
|  | <1 hour | 1.07(0.96-1.18) | 0.23 |
|  | ≥1 hour | 1.44(1.28-1.63) | <0.001 |

Abbreviation: HR, hazard ratio; BMI, body mass index.

Model adjusted for age, sex, race, household income, education, BMI, current tobacco smoking, physical activity, alcohol, sleep duration, and family history of Parkinson’s disease.

**Table S14**. Analysis of the association between daytime napping and PD with bidirectional two-sample Mendelian randomization

| **Methods** |  |  |  |
| --- | --- | --- | --- |
| **Effects of daytime napping on PD incidence** | **Number of SNPs** | **OR (95% CI)** | ***P*-value** |
| MR Egger | 93 | 1.272 (0.228 to 7.107) | 0.785 |
| Weighted median | 93 | 0.679 (0.357 to 1.289) | 0.236 |
| Inverse variance weighted | 93 | 0.816 (0.510 to 1.304) | 0.395 |
| (Multiplicative random effects model) | 93 |  |  |
| Simple mode | 93 | 0.638 (0.127 to 3.198) | 0.586 |
| Weighted mode | 93 | 0.494 (0.140 to 1.742) | 0.276 |
| MRPRESSO | 93 | 0.816 (0.510 to 1.304) | 0.397 |
| **Pleiotropy test** |  |  |  |
| MR Egger Intercept |  |  | 0.600 |
| MRPRESSO global test |  |  | 0.077 |
| **Heterogeneity test** | **Q df** | **Q** | ***P*-value** |
| IVW Cochrane Q | 112.831 | 92 | 0.069 |
| MR Egger Cochrane Q | 112.488 | 91 | 0.063 |
|  | **Value** |  |  |
| Mean F-value | 45.401 |  |  |
| **Genetic liability to PD on daytime napping** | **Number of SNPs** | **Beta (95% CI)** | ***P*-value** |
| MR Egger | 26 | 0.032 (-0.002 to 0.066) | 0.061 |
| Weighted median | 26 | -0.001 (-0.007 to 0.006) | 0.884 |
| Inverse variance weighted | 26 | 0.008 (-0.006 to 0.022) | 0.254 |
| (Multiplicative random effects model) |  |  |  |
| Simple mode | 26 | 0.002 (-0.009 to 0.013) | 0.771 |
| Weighted mode | 26 | 0.000 (-0.009 to 0.010) | 0.964 |
| MRPRESSO | 26 | 0.008 (-0.006 to 0.022) | 0.265 |
| **Pleiotropy test** |  |  |  |
| MR Egger Intercept |  |  | 0.117 |
| MRPRESSO global test |  |  | **<0.001** |
| **Heterogeneity test** | **Q df** | **Q** | ***P*-value** |
| IVW Cochrane Q | 249.253 | 25 | **<0.001** |
| MR Egger Cochrane Q | 224.522 | 24 | **<0.001** |
|  | **Value** |  |  |
| Mean F-value | 56.983 |  |  |

Bold represents *P* < 0.05

**Table S15**. F-statistics for individual instruments in two-sample Mendelian randomisation analysis

| SNP | F- statistics value |
| --- | --- |
| Daytime napping as exposure | |
| rs1001817 | 40.786 |
| rs10149986 | 48.784 |
| rs10152428 | 32.474 |
| rs10257273 | 39.694 |
| rs10811438 | 33.719 |
| rs10835420 | 40.276 |
| rs10840017 | 36.450 |
| rs10875606 | 31.824 |
| rs10875622 | 72.454 |
| rs11071755 | 33.550 |
| rs112520848 | 31.483 |
| rs11258652 | 52.957 |
| rs11615756 | 217.432 |
| rs11682175 | 32.712 |
| rs11860072 | 52.459 |
| rs12042846 | 32.141 |
| rs12140153 | 136.466 |
| rs12346996 | 34.036 |
| rs12451365 | 49.943 |
| rs12657723 | 39.716 |
| rs12992648 | 32.301 |
| rs13033444 | 52.059 |
| rs13150944 | 42.339 |
| rs13284688 | 101.101 |
| rs140506252 | 30.896 |
| rs1546977 | 45.789 |
| rs1601440 | 45.316 |
| rs17158413 | 41.869 |
| rs17265513 | 36.110 |
| rs174541 | 61.227 |
| rs17502738 | 32.366 |
| rs1883048 | 41.415 |
| rs1931175 | 38.722 |
| rs2033103 | 35.373 |
| rs2099810 | 40.114 |
| rs2143792 | 33.422 |
| rs2202323 | 36.385 |
| rs224111 | 40.855 |
| rs2250377 | 105.682 |
| rs2284015 | 30.930 |
| rs2370926 | 41.418 |
| rs253666 | 32.259 |
| rs2653349 | 126.009 |
| rs2699869 | 31.007 |
| rs2769916 | 44.414 |
| rs2786547 | 45.988 |
| rs2943023 | 33.330 |
| rs295278 | 40.209 |
| rs34262487 | 37.978 |
| rs35011311 | 43.156 |
| rs35039375 | 41.819 |
| rs350785 | 43.861 |
| rs351776 | 38.290 |
| rs35851551 | 30.511 |
| rs3799380 | 34.586 |
| rs3810484 | 31.344 |
| rs3935190 | 42.904 |
| rs4511908 | 32.032 |
| rs4604518 | 32.051 |
| rs4653052 | 30.224 |
| rs467897 | 53.956 |
| rs4692709 | 34.918 |
| rs60222088 | 42.528 |
| rs60920123 | 38.749 |
| rs614987 | 77.839 |
| rs62189006 | 33.225 |
| rs6452787 | 40.343 |
| rs6665690 | 32.743 |
| rs6919087 | 67.579 |
| rs73817091 | 29.852 |
| rs7422655 | 32.303 |
| rs7423968 | 38.808 |
| rs75022160 | 31.213 |
| rs76257331 | 32.508 |
| rs76824303 | 32.249 |
| rs7697461 | 34.423 |
| rs77154532 | 36.666 |
| rs7814873 | 32.542 |
| rs785145 | 32.535 |
| rs7932966 | 37.348 |
| rs80163246 | 39.966 |
| rs908442 | 69.196 |
| rs910187 | 33.946 |
| rs9287862 | 32.428 |
| rs9309116 | 33.613 |
| rs936944 | 30.887 |
| rs9389556 | 37.032 |
| rs9460110 | 34.215 |
| rs9475168 | 35.257 |
| rs962247 | 42.266 |
| rs971415 | 35.733 |
| rs9939355 | 35.198 |
| rs9965170 | 123.180 |
| Parkinson’s disease as exposure | |
| rs1028594 | 39.325 |
| rs10513789 | 125.220 |
| rs10756905 | 47.542 |
| rs11060180 | 40.664 |
| rs11728344 | 69.373 |
| rs12278050 | 33.397 |
| rs144814361 | 46.537 |
| rs16878376 | 31.709 |
| rs2102808 | 60.648 |
| rs2251086 | 47.987 |
| rs2736989 | 113.483 |
| rs274054 | 44.750 |
| rs3104776 | 30.323 |
| rs34096562 | 60.738 |
| rs35603727 | 95.345 |
| rs36047693 | 98.874 |
| rs3785628 | 30.763 |
| rs4588066 | 56.081 |
| rs4698413 | 67.320 |
| rs57650567 | 36.567 |
| rs59559279 | 80.182 |
| rs62300825 | 38.177 |
| rs6689008 | 60.623 |
| rs75505347 | 36.361 |
| rs9268627 | 41.879 |
| rs9933843 | 47.692 |

**Table S16**. SNPs that failed the Steiger test or identified as outliers in MR-PRESSO and Radial-MR.

|  | Methods | | | |
| --- | --- | --- | --- | --- |
|  | Steiger test | MR-PRESSO | Radial-MR |  |
| MR analysis of daytime napping on PD incidence | - | - | - |  |
| MR analysis of genetic liability to PD on daytime napping | rs36047693 | rs2102808 | rs3785628 |  |

‘-’ means no abnormal SNP detected.

**Table S17**. Sensitivity analysis of the two-sample Mendelian randomization method on the association between daytime napping and Parkinson's disease after exclusion of outlier SNPs

| **Methods** |  |  |  |
| --- | --- | --- | --- |
| **Genetic liability to PD on daytime napping** | **Number of SNPs** | **Beta (95% CI)** | ***P*-value** |
| MR Egger | 23 | 0.004 (-0.009 to 0.016 | 0.571 |
| Weighted median | 23 | -0.001 (-0.008 to 0.006) | 0.745 |
| Inverse variance weighted | 23 | -0.003 (-0.008 to 0.002) | 0.248 |
| (Multiplicative random effects model) | 23 |  |  |
| Simple mode | 23 | 0.002 (-0.010 to 0.014) | 0.782 |
| Weighted mode | 23 | 0.000 (-0.010 to 0.010) | 0.932 |
| MRPRESSO | 23 | -0.003 (-0.008 to 0.002) | 0.261 |
| **Pleiotropy test** |  |  |  |
| MR Egger Intercept |  |  | 0.272 |
| MRPRESSO global test |  |  | 0.407 |
| **Heterogeneity test** | **Q df** | **Q** | ***P*-value** |
| IVW Cochrane Q | 23.737 | 22 | 0.361 |
| MR Egger Cochrane Q | 22.380 | 21 | 0.378 |
|  | **Value** |  |  |
| Mean F-value | 56.142 |  |  |

**Figure S1** Flow chart of the screening process for this study


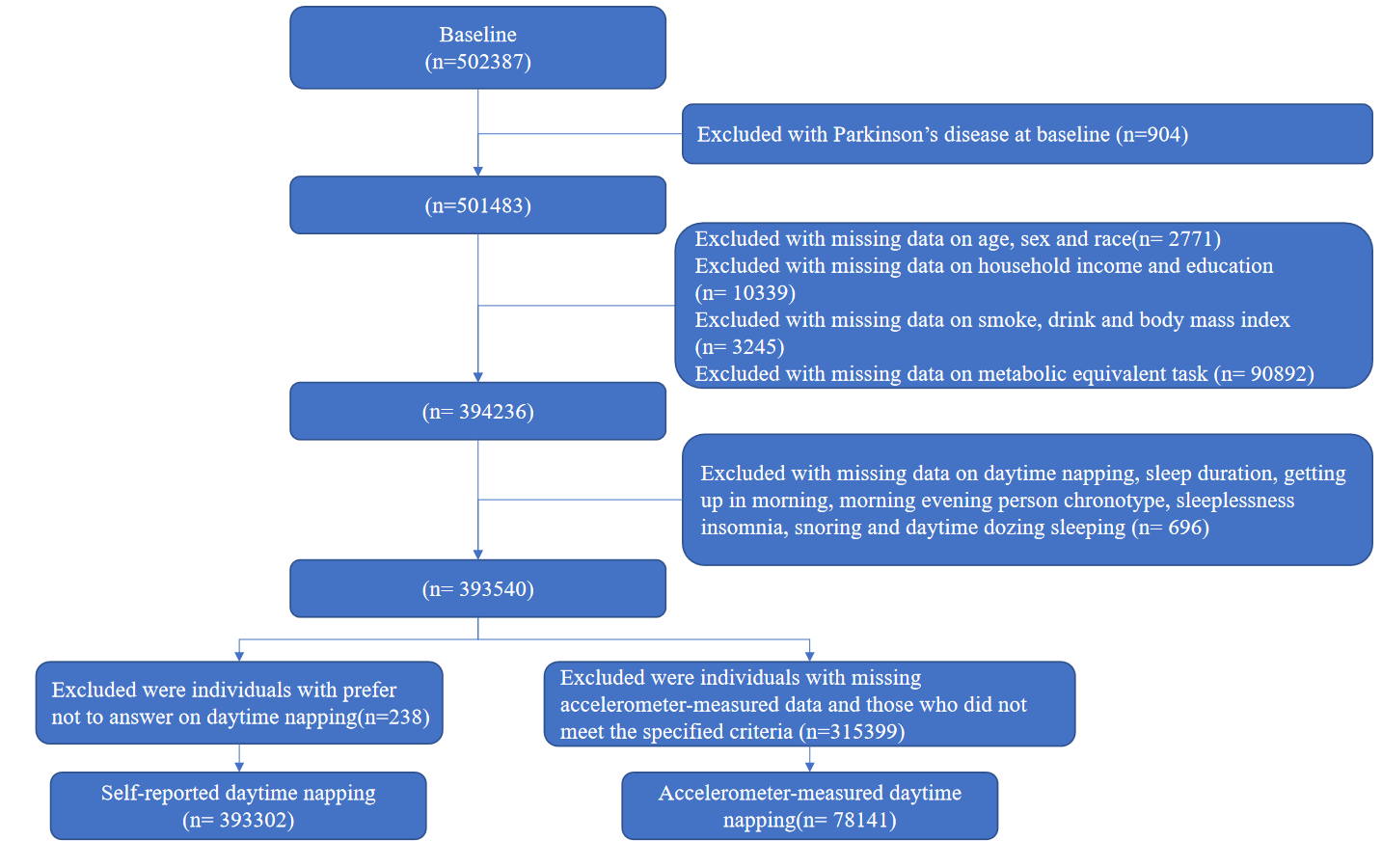


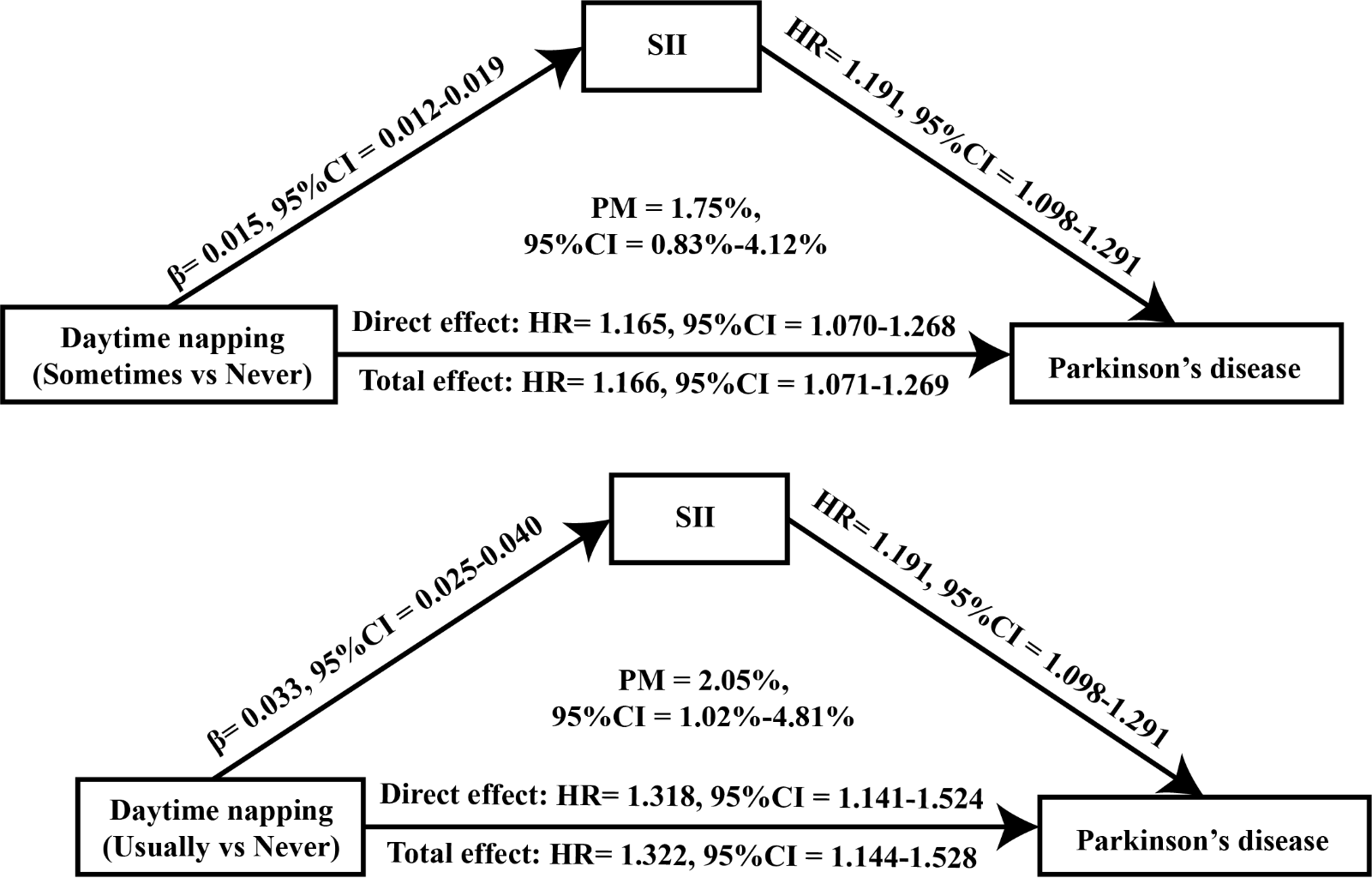


**Figure S4**. Mediation analysis of the role of systemic immune-inflammation index (SII) in the association between nap frequency and the development of PD. SII was Ln transformed in the analyses. Model adjusted for age, sex, body mass index, smoking status, alcohol consumption status, and physical activity.
