# Supplementary figures and images for: Daytime napping and the incidence of Parkinson’s disease: a prospective cohort study with Mendelian randomization
Source: BMC Med. 2024 Aug 13;22:326. doi: 10.1186/s12916-024-03497-7 (PMC11321229; doi:10.1186/s12916-024-03497-7)

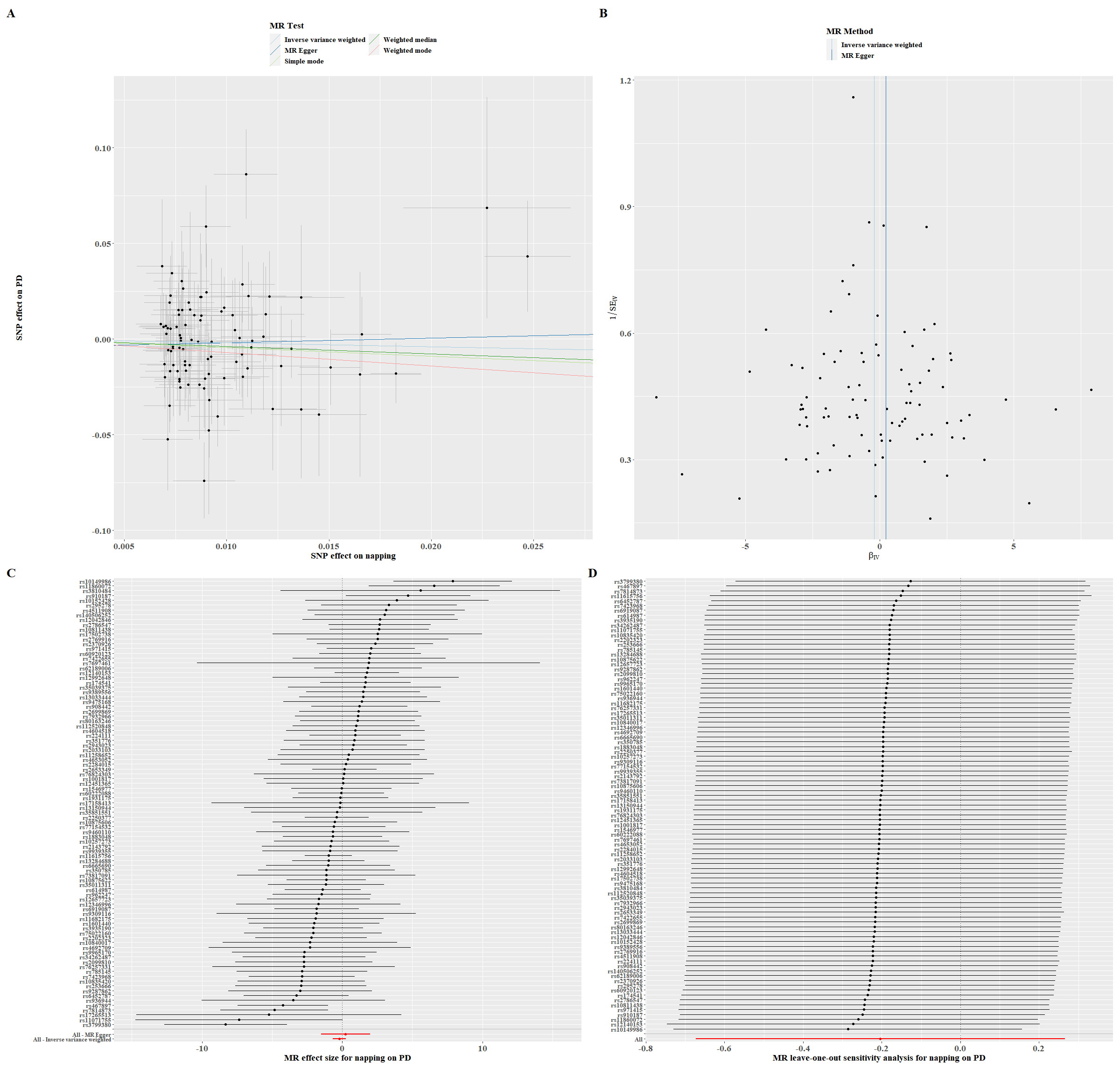

Supplement: Supplementary file 3 — Additional file 3: Fig S2- [(A) Scatter plots of genetic associations with daytime napping against the incidence of PD. (B) Funnel plot to assess heterogeneity. The blue line represents the inverse-variance weighted estimate, and the dark blue line represents the Mendelian randomization-Egger estimate. (C) Forest plot of the causal effects of single nucleotide polymorphisms associated with daytime napping on PD. The red lines are MR results of MR-Egger test and IVW method. (D) MR leave-one-out sensitivity analysis for daytime napping frequency on PD. Each black point represents the IVW-MR method applied to estimate the causal effect of daytime napping frequency on PD excluding that particular variant from the analysis]. [file 12916_2024_3497_MOESM3_ESM.tiff]

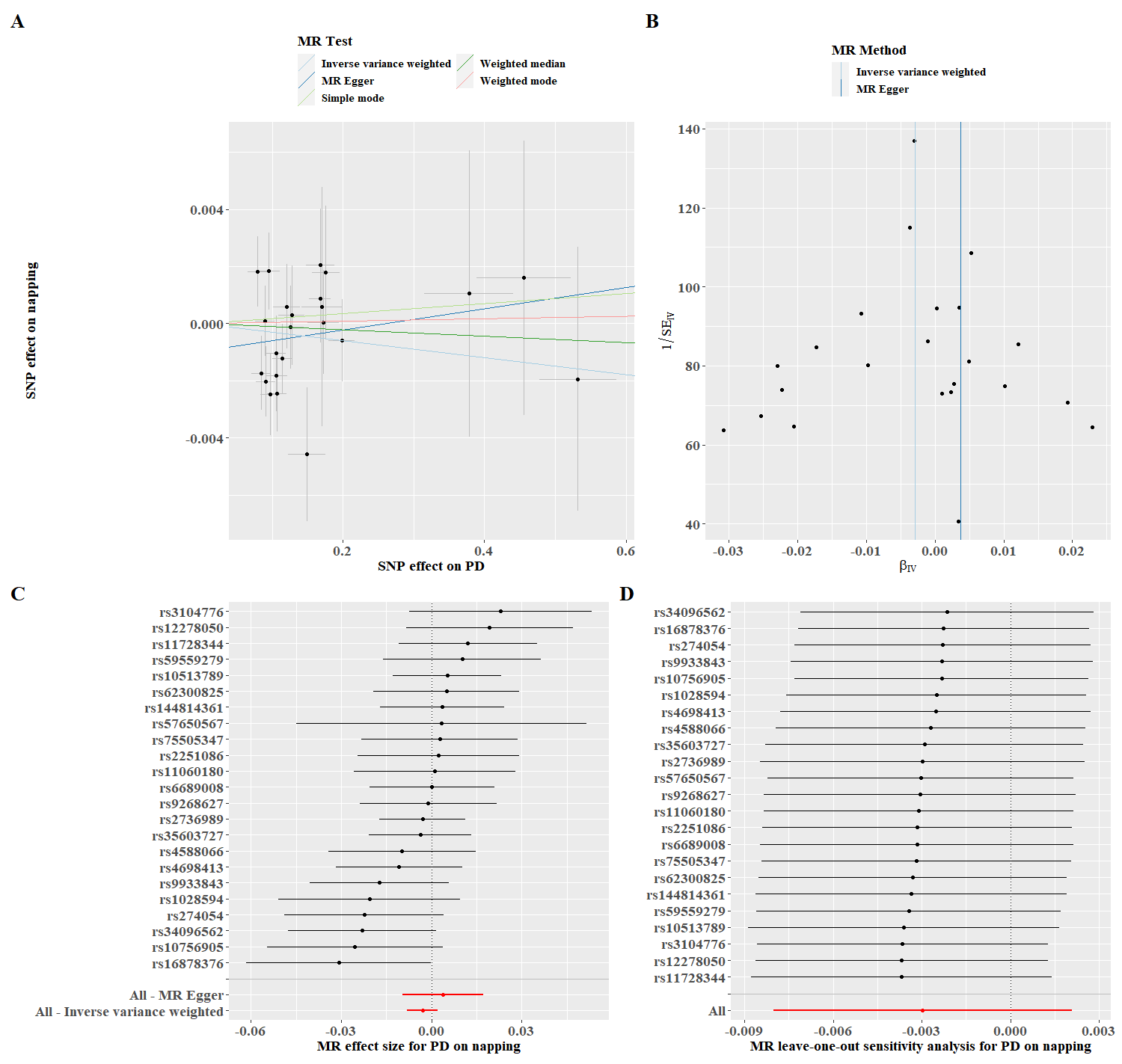

Supplement: Supplementary file 4 — Additional file 4: Fig S3- [(A) Scatter plots of genetic associations with genetic liability to PD on daytime napping. (B) Funnel plot to assess heterogeneity. The blue line represents the inverse-variance weighted estimate, and the dark blue line represents the Mendelian randomization-Egger estimate. (C) Forest plot of the causal effects of single nucleotide polymorphisms associated with genetic liability to PD on daytime napping. The red lines are MR results of MR-Egger test and IVW method. (D) MR leave-one-out sensitivity analysis for genetic liability to PD on daytime napping. Each black point represents the IVW-MR method applied to estimate the causal effect of genetic liability to PD on daytime napping excluding that particular variant from the analysis]. [file 12916_2024_3497_MOESM4_ESM.tiff]
